# An Axially Chiral Pyridyno-allenophane in a 2:1 Edge-to-Face Silver(I) Sandwich Complex: Synthesis and Structural Characterization

**DOI:** 10.1021/acsorginorgau.6c00012

**Published:** 2026-06-22

**Authors:** Jonathan Álvarez-García, María Magdalena Cid

**Affiliations:** † Departamento de Química Orgánica, Ciencias Experimentais, Universidade de Vigo, Vigo E-36310, Spain; ‡ Departamento de Química Orgánica, Universidade de Santiago de Compostela, Santiago de Compostela 15782, Spain

**Keywords:** chiral allenes, silver, cyclophanes, edge-to-face complexes, pyridyno-silver complexes, chiral pyridine-silver complexes

## Abstract

We synthesized and characterized silver­(I) complexes featuring chiral pyridyno-allenophanes—a class of cyclophanes integrating pyridine, alkyne, and allene units within a macrocyclic framework. X-ray diffraction revealed a 1:1 (ligand:metal) complex stabilized by a rare N···Ag···OH_2_···N bridging motif, and a 2:1 asymmetric sandwich-type complex, representing one of the few structurally characterized examples of its kind. Complementary solution studies (ECD, NMR, and HRMS) confirmed the persistence and stability of the 1:1 complex, which exhibits pronounced chiroptical activity. These findings establish pyridyno-allenophanes as versatile, robust scaffolds for constructing dynamic, chiral metal complexes with promising potential in supramolecular chemistry.

## Graphical abstract

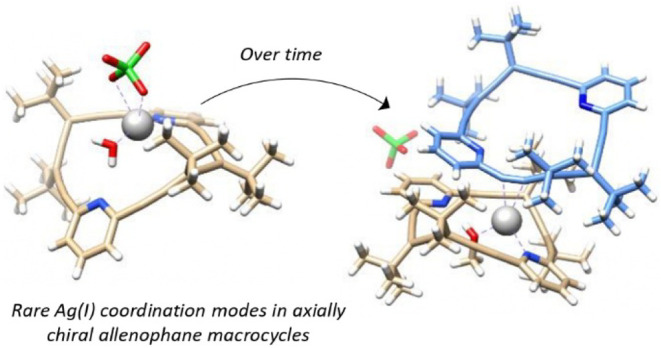

The design of novel macrocycles for metal coordination has attracted significant interest, as these architectures enable a broad range of advanced functions.^1^ In asymmetric catalysis, a chiral macrocyclic ligand forms a well-defined chiral pocket around the metal center, enhancing both catalytic activity and enantioselectivity.^2,3^ In materials science, metal–macrocycle complexes serve as versatile building blocks for tunable porous frameworks, with adjustable pore sizes suitable for molecular separations^4^ or selective uptake.^5,6^ These systems are also being adapted into chemical and biosensors capable of detecting target molecules or ions through colorimetric^7,8^ or circular dichroism responses.^9^ By broadening the scope of metal–macrocycle interactions, chemists can develop supramolecular systems with enhanced control and functional diversity, inspired by biological systems.^10^

Crown ether derivatives are widely studied macrocycles known for their specific cavity sizes and strong affinity for metal cations.^11^ Besides the common 1:1 ligand-to-metal complexes, crown ethers can also form 2:1 complexes, where two macrocycles simultaneously coordinate a single metal ion, creating sandwich-type structures.^12^ These 2:1 complexes are classified based on how the macrocycles coordinate with the metal (Scheme 1): face-to-face (type **A**), edge-to-edge (type **B**), or edge-to-face (type **C**).^13^ Type **C** complexes are the most challenging to isolate due to their asymmetric coordination, where one macrocycle binds through its cavity (*endo*) and the other through peripheral donor atoms (*exo*). Consequently, examples of type **C** remain extremely rare in the literature. The structures reported to date, summarized in Scheme 1, mainly involve Pb­(II) and Ag­(I) as metallic centers.^13,14^

**Scheme 1:**
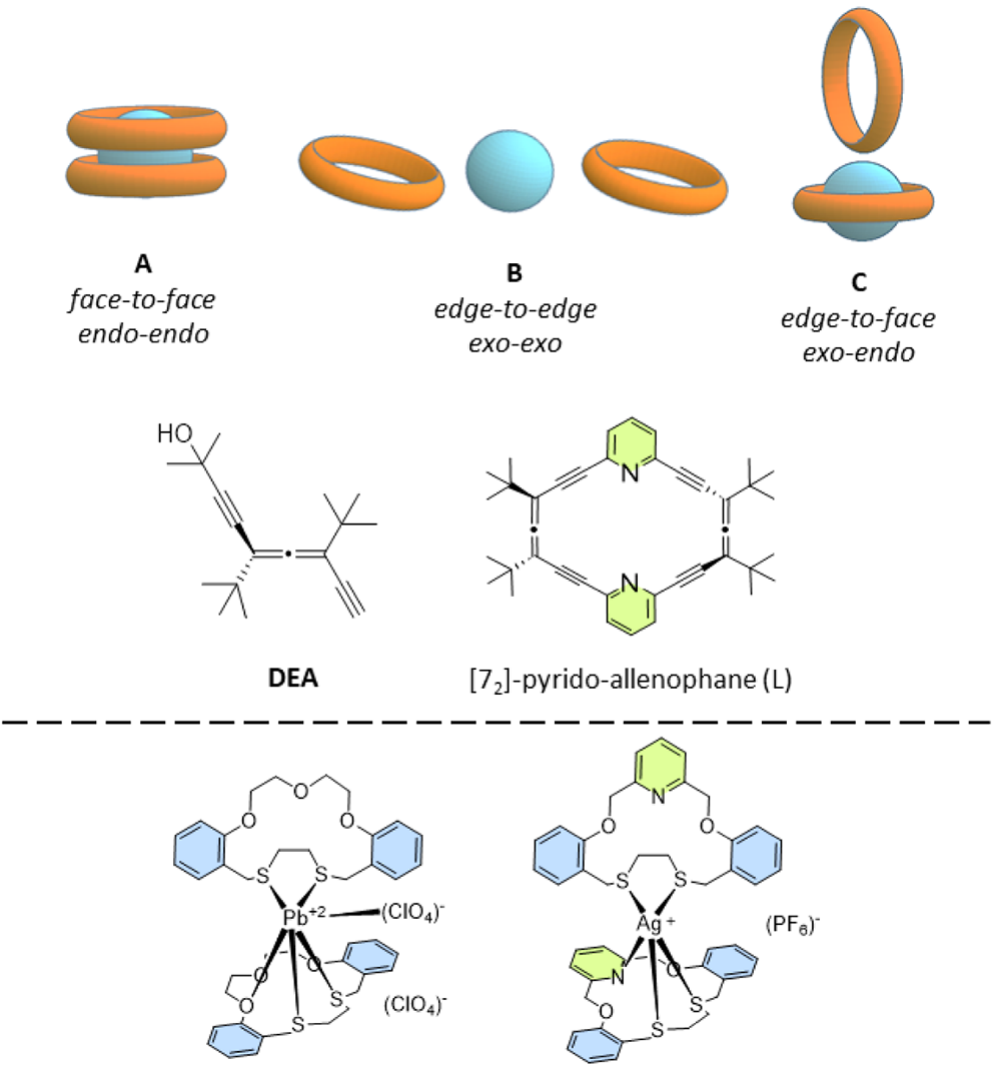
Sandwich-Type Complexes Featuring a 2:1 Ligand-to-Metal Stoichiometry^a^

a Structure of [7_2_]-pyridyno-allenophane (L). Structure of reported 2:1 *edge-to-face* complexes.

Our group focuses on the synthesis of axially chiral cyclophanes (Scheme 1, pyridyno-**allenophanes**), which provide a uniquely rigid scaffold that establishes a well-defined three-dimensional chiral environment, minimizing conformational flexibility while maximizing chiroptical responsiveness.^15,16^

We specifically utilize 1,3-diethynylallenes (Scheme 1, **DEA**s) as key building blocks.^17^ By embedding this stereogenic allene moiety within a cyclic framework, the macrocycles maintain stable axial chirality, allowing for effective and robust transfer of chiral information during guest molecule binding, while ensuring high configurational stability.^18–20^ The combination of **DEA**s with aromatic rings that act as ligands in metal coordination enables the creation of diverse supramolecular structures, inaccessible otherwise.^21–24^ However, examples of such systems remain scarce, particularly those involving macrocycles.

The coordination chemistry of Ag­(I) is notably diverse, with pyridyl nitrogen atoms serving as useful ligands.^25–27^ Alkyne–silver interactions are also of growing interest due to their distinctive geometries and potential applications, as they are promising building blocks for the construction of metallo-supramolecular polymeric architectures and luminescent materials.^28,29^ Both pyridine and alkyne moieties are present in our allenophanic structures, leading us to envision that the introduction of Ag­(I) cations could give rise to unique supramolecular architectures.

Here, we present the preparation, characterization, and structural analysis of two new complexes based on [7_2_]-pyridyno-allenophane units and silver­(I) (Scheme 1). The first complex features a 1:1 ligand-to-metal stoichiometry, in which the metal center is coordinated to one of the pyridine units within the macrocyclic cavity, while a water molecule bridges it to the second pyridine. In contrast, the second complex adopts an unsymmetrical sandwich-type **C** structure with a 2:1 stoichiometry. It displays an uncommon binding mode, where the silver ion is *endo*-coordinated within the cavity of one allenophane via a pyridine ring and a bridging methanol molecule, while simultaneously engaging in *exo*-coordination with a second allenophane through one of its alkyne units.

## Synthesis and X-ray Characterization

[7_2_]-Pyridyno-allenophane (**L**) was prepared as previously described, both in enantiopure form and as a mixture of stereoisomers.^19,30^ The stereoisomeric mixture of **L** was reacted with one equivalent of AgClO_4_ in a CHCl_3_/MeOH mixture under open-air conditions for 1 h (Scheme 2). Subsequent addition of heptane and slow evaporation of the solution afforded the complex **1a** [Ag­(**L**)­(H_2_O)­ClO_4_] in 48% yield as crystals suitable for X-ray analysis. Similar results are obtained when acetone is used as the solvent and allowed to evaporate slowly. Moreover, when the reaction is carried out under an inert N_2_ atmosphere and crystallization is attempted under the same conditions, no crystals suitable for X-ray diffraction are obtained. Only after reopening the system to air, and consequently exposing it to atmospheric moisture, can the crystallization be reproduced.

**Scheme 2:**
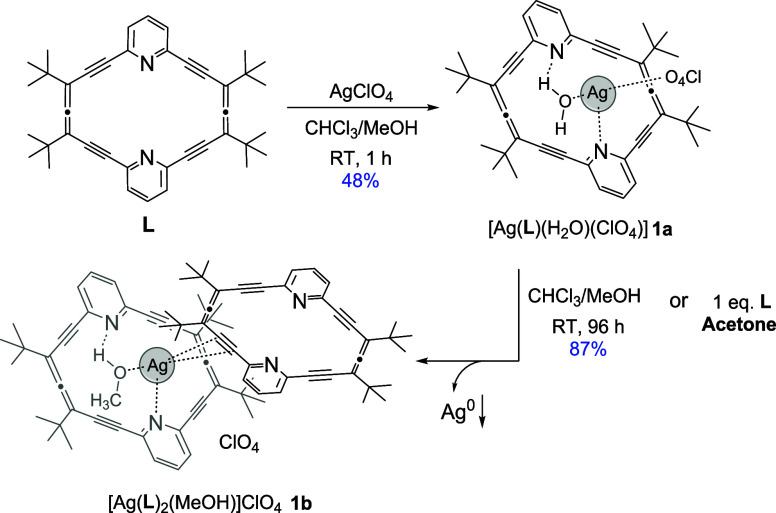
Synthesis of the Silver­(I) Complexes 1a and 1b from Ligand L

X-ray diffraction analysis revealed that complex (±)-**1a** crystallizes in the P-1 space group. The structure (Figures 1a and S4, CCDC 2465075) shows that the silver cation is located within the cavity of the allenophane, coordinated to one of the pyridine rings (N_py_···Ag, 2.16 Å) and to a water molecule (H_2_O···Ag, 2.19 Å). Remarkably, this water molecule adopts a bridging role by forming a strong hydrogen bond with the second pyridine ring of the allenophane (N_py_···HO, 1.95 Å). This results in an **N**_**py**_ **···Ag···OH**_**2**_ **···N**_**py**_ bridging motif, which is not commonly observed within the confined environment of a macrocyclic cavity. This interaction not only contributes to the stabilization of the complex but also promotes the preorganization of the allenophane framework. To the best of our knowledge, such a bridging mode—where a single water molecule links metal coordination with intracavity hydrogen bonding—has not been observed in macrocyclic systems. The perchlorate anion completes the coordination sphere of the metal center, with an average O···Ag distance of 2.72 Å.

**Figure 1:**
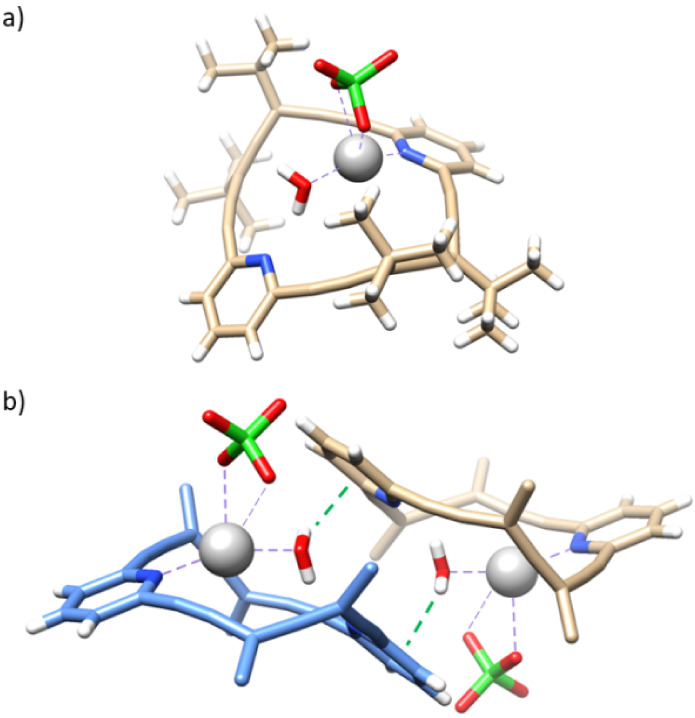
a) Crystal structure of complex **1a** obtained by X-ray diffraction. b) Supramolecular racemic dimer: in gold, allenophane (**L**) *P,P*; in blue, *M,M*. *Tert*-butyl groups were omitted for clarity. The OH···π interactions (2.95 Å) responsible for dimer formation are highlighted in green.

In addition, each **1a** complex assembles into racemic supramolecular dimers (Figures 1b and S5). These dimers are stabilized by OH···π interactions^31^ between the coordinated water molecules and the pyridine rings of neighboring complexes (π···HO, 2.95 Å). The social self-sorting^32^ behavior of these dimers is crucial for crystallization under the studied conditions, as no crystals are obtained when the enantiopure allenophanic ligand is used under the same conditions.

Subsequently, complex **1a** was redissolved in a CHCl_3_:MeOH (50:50) mixture and allowed to slowly concentrate under open-air conditions at room temperature (Scheme 2). After 48 h, a black suspension of Ag^0^ formed, which was removed by filtration, leaving a clear solution that was further concentrated for an additional 48 h. After this time, new crystals were obtained from the solution and collected by filtration (87% yield). X-ray diffraction analysis identified these crystals as the asymmetric complex **1b** (Figures 2 and S8, CCDC 2465077), consisting of two allenophane molecules coordinated to a single metal center [Ag­(**L**)_2_(MeOH)]­ClO_4_. We later found that the same complex **1b** could also be obtained by adding an additional equivalent of ligand **L** to the CHCl_3_/MeOH solution of **1a** and allowing the mixture to stand at room temperature, without the need for prior formation and removal of Ag^0^.

**Figure 2:**
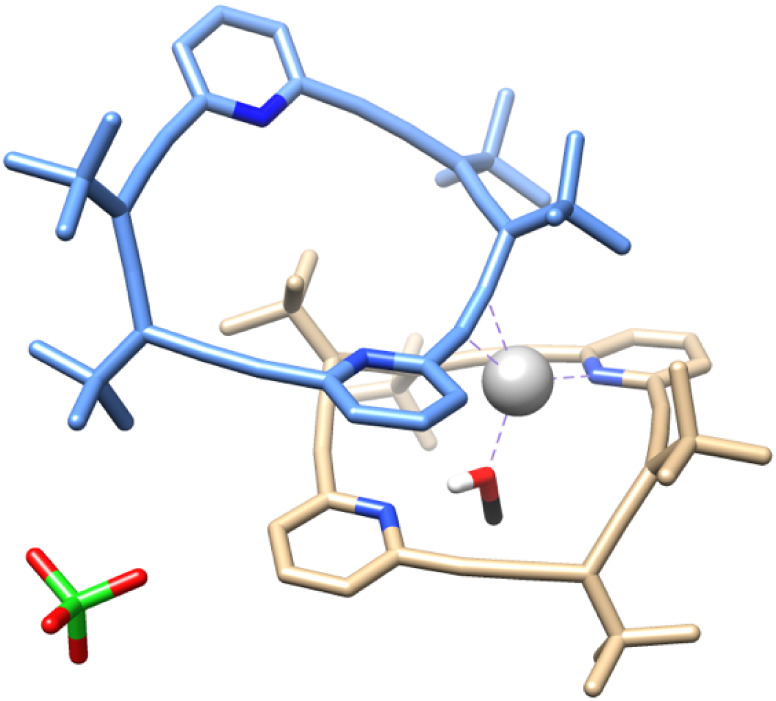
Crystal structure of complex **1b** obtained by X-ray diffraction. In gold, allenophane (**L**) *P*_,_ *P*; in blue, *M*,*M*. Hydrogen atoms were omitted for clarity.

X-ray diffraction analysis revealed that complex **1b** crystallizes in the *P*2_1_/*c* space group. The new crystal structure shows that the silver cation is located within the cavity of one of the allenophanes (*endo*-binding), coordinated to the nitrogen atom of one of its pyridine rings (N_py_···Ag, 2.2358(16) Å). A methanol molecule acts as a bridge, coordinating to the silver center (Ag···OH, 2.2104(16) Å) while simultaneously forming a strong hydrogen bond with the second pyridine ring of the same allenophane (N_py_···HO, 1.9239(16) Å). This results in an **N**_**py**_ **···Ag···OH···N**_**py**_ bridging motif very similar to that observed in complex **1a**, but with the water molecule replaced by methanol.

The second allenophane ligand coordinates to the silver center in an *exo* fashion, through one of its alkyne groups (−C≡C–···Ag, 2.3085(19) and 2.2469(19) Å), completing the metal coordination sphere. These alkyne–silver bond distances fall within the range typically observed for η^2^ -alkyne silver­(I) complexes and are comparable to those reported in related systems, where Ag–(η^2^ -C≡C) interactions generally lie between ca. 2.20 and 2.35 Å.^33–35^ In addition, the alkyne bound to the silver center exhibits a slight elongation of the C≡C bond (1.218(3) Å) relative to the other three alkyne units present in the same allenophane ligand, which display an average C≡C distance of 1.200(3) Å. This modest lengthening is consistent with π-coordination to the metal center. Furthermore, the perchlorate counteranion remains outside the inner coordination sphere. Notably, the resulting complex is composed of two pyridyno-allenophanes of opposite (*M* and *P*) configuration, mirroring the heterochiral dimerization observed for compound **1a**. This assembly yields a rare and structurally unique C-type, asymmetric sandwich complex.

Comparison of the IR spectra obtained for complexes **1a** and **1b** (Figure S2b and S2c) reveals a subtle yet significant difference in the alkyne stretching region. For complex **1a**, a single ν­(C≡C) stretching band is observed at 2206 cm^–1^. In contrast, the spectrum of complex **1b** displays, in addition to this signal, a second weaker band at 2165 cm^–1^. These absorptions can be assigned to the noncoordinated alkynes and to the alkyne unit coordinated to the silver center, respectively.

The observed shift of 41 cm^–1^ upon coordination falls within the range reported by Noonikara-Poyil and coworkers for Ag^+^ coordination to acetylene derivatives (34–79 cm^–1^)^36^ and is slightly smaller than the value reported by Schelter and coworkers for a silver­(I) tris­(alkyne) complex (66 cm^–1^).^29^ This relatively modest shift suggests a limited degree of Ag→alkyne π-backdonation, in agreement with the crystallographic data, where only a subtle elongation of the coordinated C≡C bond was observed.

To investigate whether the transformation of **1a** into **1b** is driven by thermodynamic stabilization, we employed a computational model (Scheme 3) in which the reactants consist of complex **1a**, the free macrocycle (L), and one molecule of methanol, yielding complex **1b** and water as products. Both reactant and product species were fully optimized using the Gaussian package^37^ at the B3LYP-D3 ^38^/def2-TZVP level of theory.^39–42^ The computed Gibbs free-energy change (ΔG) for this model is positive (+50.0 kcal mol^–1^), indicating that the 1:1 → 2:1 complex conversion is thermodynamically unfavorable under the studied conditions. Instead, these results suggest that the experimentally observed formation of **1b** is likely governed by factors not captured in the model, such as changes in effective concentrations under the experimental conditions.

**Scheme 3:**
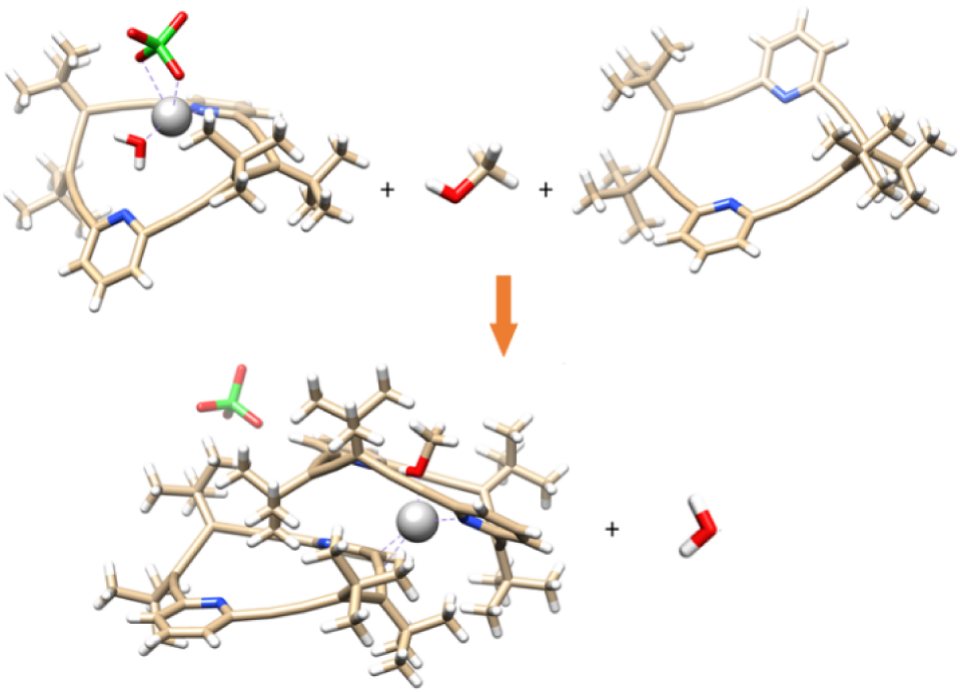
Computational Model of the 1a → 1b Transformation

As solutions of complex **1a** stand and undergo partial solvent evaporation, a fraction of the silver­(I) ions is reduced, leading to the formation of black Ag^0^ particles that effectively remove silver from the solution. This precipitation decreases the concentration of Ag^+^, thereby increasing the relative ligand-to-metal ratio ([L]:[Ag^+^]) in the solution and shifting the coordination equilibrium toward higher-order stoichiometries. Under these conditions, the remaining Ag^+^ centers can coordinate a second macrocycle, promoting the formation of the 2:1 complex. Subsequent crystallization then stabilizes and locks in the observed edge-to-face geometry of **1b**. A similar effect is achieved by increasing the concentration of L through the addition of extra ligand to the preformed **1a** complex.

These results demonstrate that [7_2_]-pyridyno-allenophane (**L**) supports unusual silver­(I) coordination patterns, including internal bridging motifs and asymmetric 2:1 complexes, showing its structural versatility in the solid state. Given these striking solid-state structures, we next aimed to investigate the complexation behavior of **L** with silver­(I) perchlorate in solution.

### Solution Studies

Taking advantage of the strong chiroptical responses of allenophane **L**, we performed a titration of the enantiopure ligand (*P*,*P*)-**L** with AgClO_4_, monitored by circular dichroism in a chloroform:methanol (95:5) mixture (Figures 3a and S9). As the concentration of the silver salt increases in solution, the negative Cotton effects of (*P*,*P*)-**L** initially centered at 305 and 324 nm shift to 320 and 340 nm, respectively (Figure 3a, red and blue lines, respectively). This phenomenon is consistent with the coordination of Ag^+^ to the pyridine moiety, resulting in a decrease in electron density over the ring. Simultaneously, a new positive band appears centered at 295 nm. This observed band corresponds to (*P*,*P*)-**L** allenophane adopting a concave V-shaped conformation. This conformation aligns well with prior computational models^19^ and the solid-state structure of complex **1a,** strongly suggesting a similar structural motif persists in solution. This similarity suggests that the complex in solution possesses a structure analogous to the solid-state structure of **1a**. Furthermore, the presence of two isodichroic points at 275 and 330 nm further supports the probable formation of a 1:1 stoichiometric complex. The experimental data from this titration were fitted to a 1:1 binding equilibrium using the BindFit program,^43,44^ yielding a very good fit and an association constant of (4.0 ± 0.8) × 10^5^ M^–1^ (Figure S10). Although the obtained association constant is not as high as those observed for polydentate ligands,^45^ the result is still noteworthy, considering that coordination occurs through a single pyridine unit within a structurally constrained macrocyclic scaffold. Notably, this titration leads to the formation of a chiral silver­(I) macrocyclic complex exhibiting a strong chiroptical response.

**Figure 3:**
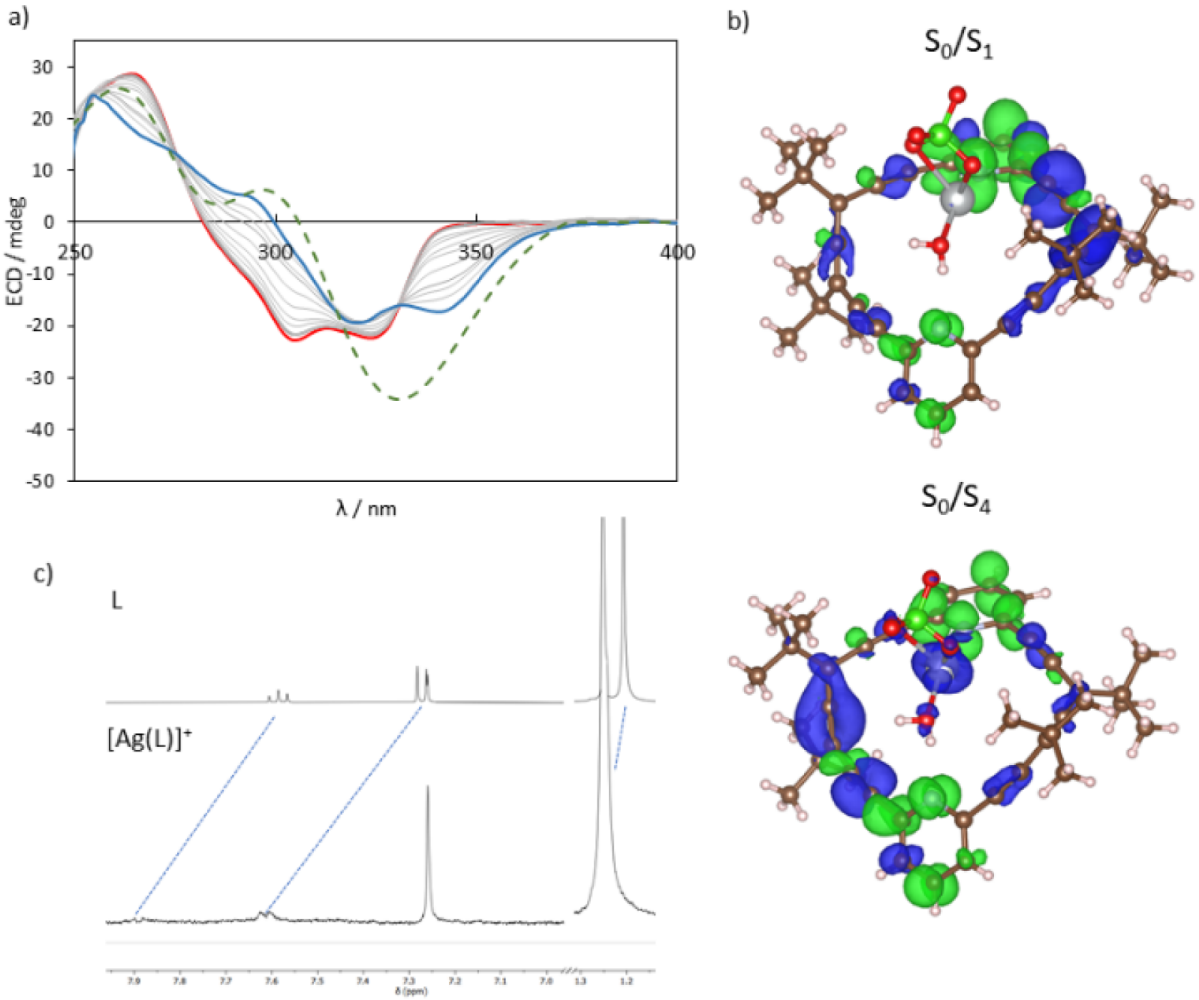
a) Titration by electronic circular dichroism (ECD) of ligand (*P*,*P*)-**L** (1.5 × 10^–5^ M, CHCl_3_:MeOH (95:5)) as the concentration of AgClO_4_ in solution increases. The red line represents the original spectrum of (*P*,*P*)-**L**, and the blue line corresponds to the spectrum at the titration end point. The green dashed line represents the ECD spectrum calculated for the geometry of complex **1a** (TD-DFT CAM-B3LYP/6–31+G­(d,p)/def2-TZVP, smd = CHCl_3_). b) Hole–electron analysis of S_0_ → S_1_ and S_0_ → S_4_ transitions. c) Change in the^1^ H NMR spectrum of **L** (0.024 M, CDCl_3_, top) upon addition of 1.1 equiv of AgClO_4_ (bottom).

Since the collected data suggest the formation of a complex in solution that is structurally similar to **1a** in the solid state, the ECD spectrum of this complex was computationally calculated and compared with the experimental one. The ECD spectrum was calculated at the TD-DFT CAM-B3LYP^46^/6–31+G­(d,p) level, using the def2-TZVP basis set with a pseudopotential on the silver atom, and including solvation effects using the SMD model^47^ with chloroform parameters (Figure 3a, green dashed line). We found that both spectra matched very well, providing further support for the proposed solution structure.

The origin of the main ECD bands was analyzed using the hole–electron method in the Multiwfn program.^48^ The negative band at 310 nm corresponds to the S_0_ → S_1_ transition. The hole density (blue) is concentrated on the π orbitals of the pyridine and alkyne fragments, while the excited electron density (green) spreads over adjacent regions of the conjugated framework, with minor involvement of the silver center (Figure 3b). This suggests a predominantly intraligand π→π* transition. On the other hand, the positive band at 275 nm arises mainly from the S_0_ → S_4_ and S_0_ → S_5_ transitions, which exhibit very similar characteristics (Figure S12). For both transitions, the hole is largely centered on the silver atom, while the excited electron density is delocalized over the ligand π-system, indicating a significant metal-to-ligand charge-transfer (MLCT) contribution (Figure 3b). The highest-energy band is described by a cluster of multiple transitions (Figure S12). These results confirm that the calculated transitions accurately reflect the electronic structure of the complex, supporting the assignments of the observed ECD bands.

Additionally, the solution resulting from the titration was analyzed by high-resolution electrospray ionization mass spectrometry (HRMS-ESI, Figure S3). The spectrum was dominated by a peak corresponding to [Ag­(**L**)]^+^ (*m*/*z* 657.2388), indicating the lability of the remaining ligands and highlighting both the dynamic nature of the coordination sphere and the potential for incorporation of additional ligands.

To further investigate the formation of complex **1a** in solution, we carried out NMR spectroscopic studies. Upon addition of 1.1 equiv of AgClO_4_ to a solution of **L** in CDCl_3_, significant changes were observed in the ^1^H NMR spectrum. The aromatic signals corresponding to the pyridine rings exhibited a pronounced downfield shift (ΔδH_d_ ≈ + 0.34 ppm) upon coordination with silver, accompanied by extensive broadening and a noticeable loss of resolution—nearly vanishing into the baseline (Figure 3c). In contrast, the signal corresponding to the *tert*-butyl groups experienced only a slight deshielding (ΔδH ≈ + 0.04 ppm) and remained well resolved, suggesting that the process primarily affects the coordination environment of the pyridine nitrogen atoms.

These spectroscopic features are consistent with a coordination equilibrium involving rapid exchange of the Ag­(I) ion between the two pyridine moieties of the macrocyclic ligand, or fast interconversion between the free and coordinated forms of the ligand on the NMR time scale. Attempts to slow down this exchange process by lowering the temperature to −15 °C did not significantly improve signal resolution. Further cooling was not feasible due to the precipitation of the complex, which resulted in a complete loss of signal.

Both the ECD and NMR solutions were reevaluated several months later to assess the stability of complex **1a** in solution, having been stored in a sealed vessel. No significant changes were observed over this period, indicating high temporal stability of the complex in solution. No evidence was found for the formation of other complexes with different stoichiometries, such as **1b**, likely due to the influence of solvation. This observation supports our previous hypothesis that solvent loss and partial silver precipitation are the key triggers for the **1a** → **1b** transformation.

Additionally, when single crystals of **1b** were dissolved in CDCl_3_ with the aim of characterizing the complex by ^1^H NMR spectroscopy, a highly complex mixture of species was observed, displaying numerous broadened and overlapping aromatic resonances (Figure S1c). In contrast to the relatively simple spectral pattern obtained for **1a**, the spectrum of dissolved **1b** lacked a well-defined set of signals attributable to a single discrete species. This behavior further demonstrates that the solid-state structure of **1b** is not retained in solution, where the system becomes highly fluxional and sensitive to changes in concentration, solvation, and metal-to-ligand ratio.

A similar phenomenon was reported by Park and coworkers, where an asymmetric Pb­(II) sandwich-type complex was not formed in solution but was only observed in the solid state. The authors attributed this behavior to the strong solvation of the metal center by polar solvents such as acetonitrile, which stabilizes the metal ion in solution and hinders the coordination of a second macrocycle. As a consequence, the coordination process remains limited to the formation of a 1:1 metal–macrocycle complex in solution, whereas the 2:1 sandwich-type structure becomes accessible only upon crystallization.^13^ These observations further support the notion that solvent-dependent solvation and crystallization processes can play a decisive role in determining the final coordination motif in macrocyclic metal complexes.

## Conclusions

In summary, we have demonstrated that [7_2_]-pyridyno-allenophane (**L**), a rigid axially chiral macrocycle incorporating pyridine and alkyne functionalities, supports the formation of structurally unique silver­(I) complexes. Two distinct architectures were obtained and characterized by X-ray diffraction: a mononuclear 1:1 complex (**1a**), stabilized by a water-bridged N_py_···Ag···OH_2_···N_py_ motif within the macrocyclic cavity, and a rare asymmetric 2:1 sandwich-type complex (**1b**) featuring simultaneous *endo*- and *exo*-coordination of two allenophane ligands via pyridine and alkyne groups, respectively.

These solid-state structures reveal the remarkable preorganization and coordination versatility of the pyridyno-allenophane scaffold, including its ability to support unusual internal bridging interactions and promote the formation of supramolecular dimers via OH···π contacts.

Complementary studies in solution using ECD, NMR spectroscopy, and HRMS provided evidence for the persistence of complex **1a** in a dynamic equilibrium. This equilibrium is characterized by strong chiroptical responses and high temporal stability under ambient conditions. While complex **1b** was not observed in solution, its exclusive formation in the solid state highlights the critical role of solvation and crystallization conditions in driving and stabilizing these asymmetric assemblies.

Altogether, these findings underscore the potential of pyridyno-allenophanes as robust, chiral scaffolds for generating responsive and architecturally diverse metal complexes. Their capacity to engage in *endo*- and *exo*-coordination, combined with pronounced chiroptical properties and dynamic solution behavior, makes them attractive candidates for further exploration in supramolecular chemistry, catalysis, and molecular recognition.

## Experimental Methods

### General Information

^1^H NMR spectra were recorded at 25 °C (unless otherwise stated) on Bruker AMX-400 at 400 MHz with residual protic solvent as the internal reference [CDCl_3_, δ_H_ = 7.26 ppm]. Chemical shifts (δ) are given in parts per million (ppm), and coupling constants (J) are given in Hertz (Hz). The proton spectra are reported as follows: chemical shift δ (multiplicity, coupling constant *J*, number of protons). The following symbols were used for the description of coupling patterns: broad (br), multiplet (m), singlet (s), doublet (d), triplet (t). ^13^C NMR spectra were recorded on the same spectrometer at 100 MHz at 25 °C (unless otherwise stated), with residual protic solvent as the internal reference [CDCl_3_, δ = 77.16 ppm]. ECD and UV–vis spectra were recorded on a Jasco J-815 spectropolarimeter using a one-centimeter quartz cuvette at 25 °C. ESI mass spectra were recorded with an APEX3 instrument. Ions were generated using a Combi MALDI–electrospray ionization (ESI) source. High-resolution mass spectra were taken on a VG Autospec instrument. Crystallographic data were collected at 100 K using a Bruker D8 Venture diffractometer with a Photon II CMOS detector and Mo–Kα radiation (λ = 0.71073 Å) generated by an Incoatec high-brilliance microfocus source equipped with Incoatec Helios multilayer optics. Excited-state energies and properties were computed using TD-DFT at the CAM-B3LYP^46^/6–31+G­(d,p) level of theory. For the silver atom, the def2-TZVP^39^ basis set with a pseudopotential was employed, obtained from the Basis Set Exchange library.^42^ Solvation effects were included using the SMD^46^ model with chloroform as the solvent. The first 30 excited states were calculated.

### Complex [Ag­(L)­(H_2_O)­(ClO_4_)] 1a

A mixture of stereoisomers of [7_2_]-pyrido-allenophane (**L**)^11^ (20 mg, 0.036 mmol, 1 equiv) was dissolved in 4 mL of a CHCl_3_:MeOH (50:50) mixture in an amber vial. AgClO_4_ (8 mg, 0.038 mmol, 1.1 equiv) was added, and the mixture was stirred for 1 h at room temperature without the need for an inert atmosphere. The reaction mixture was filtered using a 0.45 μm syringe filter, and 1.5 mL of heptane was added. The resulting solution was allowed to evaporate slowly in open air. After 48 h, colorless crystals were obtained, which were filtered and further dried under reduced pressure. The crystals were identified as complex **1a** by single-crystal X-ray diffraction, with the molecular formula [Ag­(L)­(H_2_O)­(ClO_4_)] (13 mg, 48% yield), in which **L** is present as a racemate of its homochiral forms (*P*,*P*)-L and (*M*,*M*)-L. Enantiopure (*P*,*P*)-**1a** was obtained following the same reaction conditions starting from (*P*,*P*)-**L**.

### Complex [Ag­(L)_2_(MeOH)]­(ClO_4_) 1b

Complex [Ag­(L)­(H_2_O)­(ClO_4_)] (**1a**) (10 mg, 0.013 mmol) was dissolved in 4 mL of a CHCl_3_:MeOH (50:50) mixture in an amber vial, and the solution was left to stand at room temperature in open air for 48 h while slowly concentrating. After this time, a black suspension of Ag^0^ had formed, which was filtered using a 0.45 μm syringe filter. The clear solution was then allowed to concentrate for an additional 48 h, yielding crystals that were identified by single-crystal X-ray diffraction as complex **1b**, with the molecular formula [Ag­(L)_2_(MeOH)]­(ClO_4_) (7.5 mg, 87% yield).

## Supplementary Material



## Data Availability

The data underlying this study are available in the published article and its Supporting Information.

## Notes

General experimental methods, including synthesis conditions, characterization techniques, titration protocols, and computational details (PDF)
