# Supplementary material for: An Axially Chiral Pyridyno-allenophane in a 2:1 Edge-to-Face Silver(I) Sandwich Complex: Synthesis and Structural Characterization
Source: ACS Org Inorg Au. 2026 Jun 22;6(4):406–13. doi: 10.1021/acsorginorgau.6c00012 (PMC13449429; doi:10.1021/acsorginorgau.6c00012)
Supplement: Supplementary file 1 [file gg6c00012_si_001.pdf]

An Axially Chiral Pyridyno-Allenophane in a 2:1 Edge-to-face Silver(I) Sandwich Complex: Synthesis and Structural Characterization

Jonathan Álvarez-García[a]*, María Magdalena Cid[b]*

[a]Departamento de Química Orgánica, Edificio Ciencias Experimentais, Universidade de Vigo, E-36310, Spain.

[b]Departamento de Química Orgánica, Universidade de Santiago de Compostela, 15782 Santiago de Compostela, Spain.

Table of Contents

1. General methods 3
2. Synthesis and characterization 4
   2.1. Crystallographic Data 9
3. ECD Titration 15
4. Simplified computational model of the 1a → 1b transformation 16
5. Hole-electron analysis 17
6. References 17

1. General methods

^1H NMR spectra were recorded at 25 ^oC (unless otherwise stated) on Bruker AMX-400 at 400 MHz with residual protic solvent as the internal reference [$CDCl_3$, δ_H = 7.26 ppm]. Chemical shifts (δ) are given in parts per million (ppm), and coupling constants (J) are given in Hertz (Hz). The proton spectra are reported as follows: chemical shift δ (multiplicity, coupling constant *J*, number of protons). The following symbols were used for the description of coupling patterns: broad (br), multiplet (m), singlet (s), doublet (d), triplet (t). ^{13}C NMR spectra were recorded on the same spectrometer at 100 MHz at 25 ^oC (unless otherwise stated) with residual protic solvent as the internal reference [$CDCl_3$, δ = 77.16 ppm]. ECD and UV-vis spectra were recorded on a Jasco J-815 spectropolarimeter using a one-centimeter thick quartz cuvette at 25 °C. The background was always obtained against the solvent. ESI mass spectra were recorded with an APEX3 instrument. Ions were generated using a Combi MALDI-electrospray ionization (ESI) source. High-resolution mass spectra were taken on a VG Autospec instrument.

Crystallographic data were collected at 100 K using a Bruker D8 Venture diffractometer with a Photon II CMOS detector and Mo-Kα radiation (λ = 0.71073 Å) generated by an Incoatec high brilliance microfocus source equipped with Incoatec Helios multilayer optics. The software APEX4[1] was used for collecting frames of data, indexing reflections, and determination of lattice parameters, SAINT[2] for integration of intensity of reflections, and SADABS[3] for scaling and empirical absorption correction. The structure was solved by dualspace algorithm using the program SHELXT.[4] All non-hydrogen atoms were refined with anisotropic displacement parameters by full-matrix least-squares calculations on F^2 using the program SHELXL[5] with OLEX2[6]. Hydrogen atoms were inserted at calculated positions and constrained with isotropic displacement.

Excited-state energies and properties were computed using TD-DFT at the CAM-B3LYP[7]/6-31+G(d,p) level of theory. For the silver atom, the def2-TZVP[8] basis set with a pseudopotential was employed, obtained from the Basis Set Exchange library.[9] Solvation effects were included using the SMD[10] model with chloroform as the solvent. The first 30 excited states were calculated.

2. Synthesis and characterization

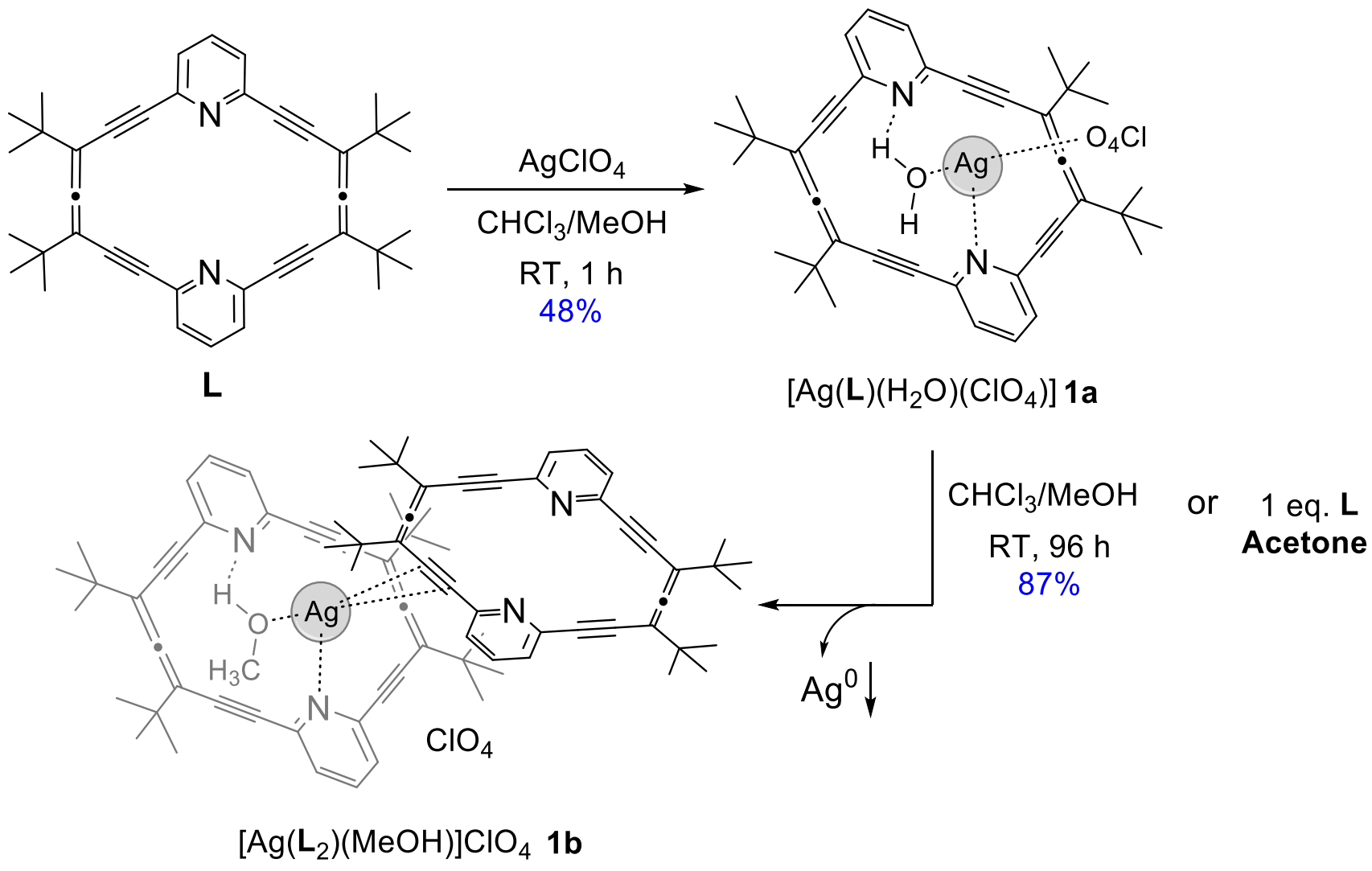

Complex [Ag(L)(H_2O)(ClO_4)] 1a

A mixture of stereoisomers of [7_2]-pyrido-allenophane (**L**)[11] (20 mg, 0.036 mmol, 1 equiv) was dissolved in 4 mL of a $CHCl_3$:MeOH (50:50) mixture in an amber vial. $AgClO_4$ (8 mg, 0.038 mmol, 1.1 equiv) was added, and the mixture was stirred for 1 h at room temperature without the need for an inert atmosphere. The reaction mixture was filtered using a 0.45 µm syringe filter, and 1.5 mL of heptane was added. The resulting solution was allowed to evaporate slowly in open air. After 48 h, colorless crystals were obtained, which were filtered and further dried under reduced pressure. The crystals were identified as complex **1a** by single-crystal X-ray diffraction, with the molecular formula [Ag(L)(H_2O)(ClO_4)] (13 mg, 48% yield), in which **L** is present as a racemate of its homochiral forms (*P*,*P*)-L and (*M,M*)-L.

As an alternative approach, different experimental conditions were explored in order to broaden the scope of the study and potentially obtain other systems. It was found that performing the same procedure using acetone as the solvent led to the formation of complex **1a** with comparable results. In contrast, when other solvents such as pyridine or acetonitrile were employed—both capable of competing for coordination at the silver center—neither **1a** nor any other identifiable product was obtained.

Additionally, the reaction in the chloroform/methanol mixture was carried out under an inert atmosphere. However, crystallization of complex **1a** was only achieved after the solution had been exposed to air for a certain period of time.

Complex [Ag(L)$_2$(MeOH)](ClO_4) 1b

Complex [Ag(L)(H_2O)(ClO_4)] (**1a**) (10 mg, 0.013 mmol) was dissolved in 4 mL of a $CHCl_3$:MeOH (50:50) mixture in an amber vial, and the solution was left to stand at room temperature in open air for 48 h while slowly concentrating. After this time, a black suspension of Ag^0 had formed, which was filtered using a 0.45 µm syringe filter. The clear solution was then allowed to concentrate for an additional 48 h, yielding crystals that were identified by single-crystal X-ray diffraction as complex **1b**, with the molecular formula [Ag(L)$_2$(MeOH)](ClO_4) (7.5 mg, 87% yield).

We found that the same complex **1b** could also be obtained by adding an additional equivalent of ligand **L** to the $CHCl_3$/MeOH solution of **1a** and allowing the mixture to stand at room temperature, without the need for prior formation and removal of Ag^0.

1a: ^{1}H NMR (400 MHz, $CDCl_3$) δ (ppm) 7.90 (br, 1), 7.61 (br, 2), 2.18 (br, H_2O, 4)[1], 1.25 (s, 3). **^{13}C NMR** (100 MHz, $CDCl_3$) δ (ppm) 215.4 (7), 131.3 (2), 128.8 (3), 126.2 (1), 104.9 (6), 77.4 (4), 68.1 (5), 35.4 (8), 29.03 (9). **UV-vis** ($CHCl_3$:MeOH (95:5)): λ_{max} (ε)= 275 nm (44156 mol^{-1} dm^3 cm^{-1}), 296 nm (38158 mol^{-1} dm^3 cm^{-1}), 315 nm (34681 mol^{-1} dm^3 cm^{-1}), 341 nm (13211 mol^{-1} dm^3 cm^{-1}). **ECD** ($CHCl_3$:MeOH (95:5)): λ_{max} (Δε)= 275 nm (|893687| mol^{-1} dm^3 cm^{-1}), 296 nm (|293851| mol^{-1} dm^3 cm^{-1}), 315 nm (|1149867| mol^{-1} dm^3 cm^{-1}), 341 nm (|1146207| mol^{-1} dm^3 cm^{-1}). **HRMS-ESI**: m/z calcd. for $C_{40}H_{42}N_2Ag^+$ 657.2393; found 657.2388 $[Ag(L)]^+$. **IR** (ν, cm^{-1}) 3633-3513 (OH), 2959-2866 (CH), 2206 (C≡C), 1619-1459 (py), 1094-1072 (Cl-O). **Anal** Calcd for $C_{40}H_{44}N_2AgClO_5$: C, 61.90; H, 5.71; N, 3.60. Found C, 61.62; H, 5.70; N, 3.58.

1b: IR (ν, cm^{-1}) 3527-3522 (OH), 2961-2904 (CH), 2206 (C≡C), 2165 (C≡C···Ag), 1619-1458 (py), 1091-1073 (Cl-O). **Anal** Calcd for $C_{81}H_{88}N_4AgClO_5$: C, 72.55; H, 6.62; N, 4.18. Found C, 72.40; H, 6.67; N, 4.13.

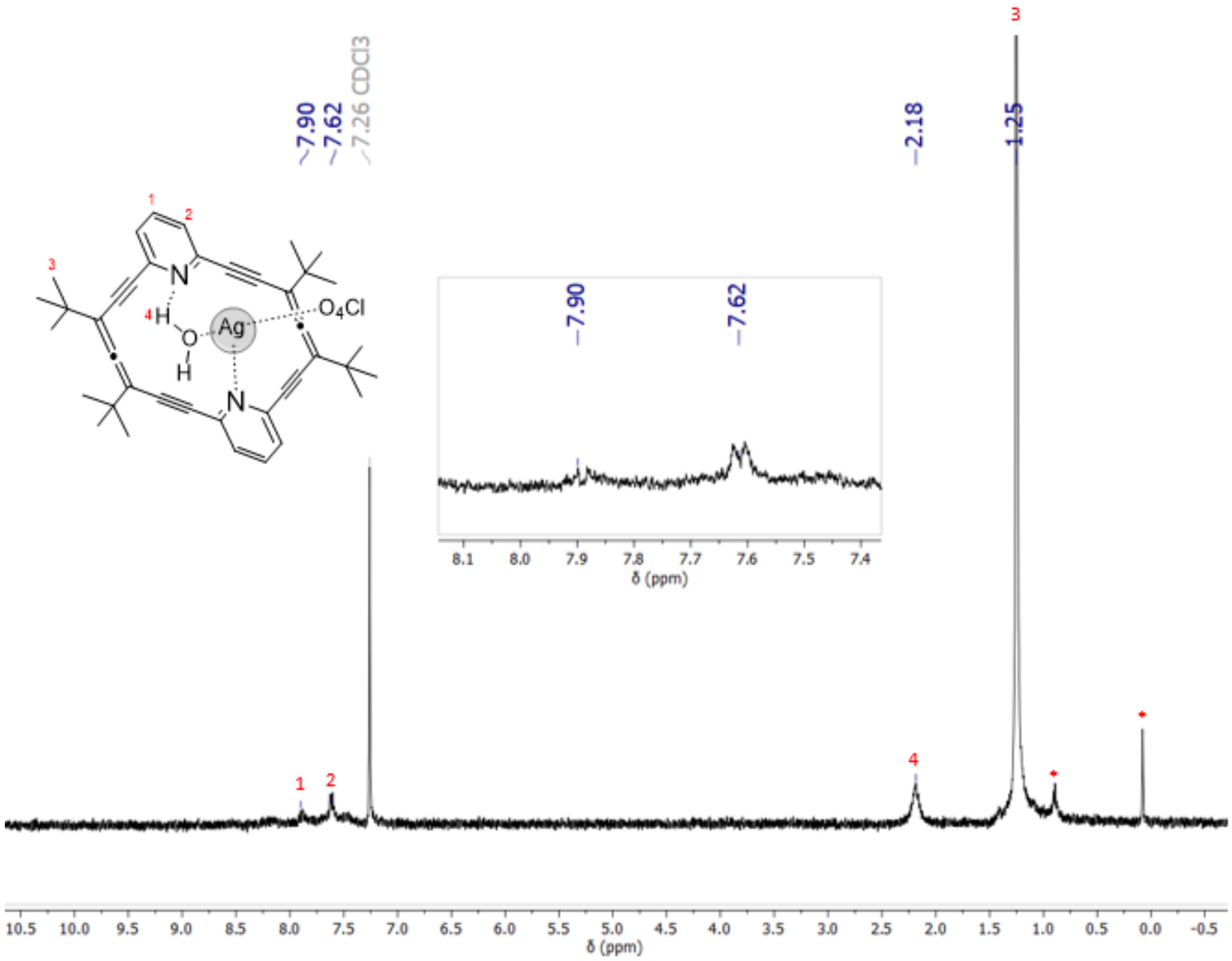

Figure S1a. *^{1}H NMR (400 MHz) spectrum of complex **1a** in $CDCl_3$. Signal integrations are omitted due to significant line broadening, which prevents reliable integration. Signals marked with an asterisk (*) correspond to impurities.*

[1] The resonance assigned to the coordinated water molecule appears significantly downfield compared to free water. This deshielding is attributed to coordination of the water molecule to the metal center and pyridine. The broad nature of this signal is also consistent with exchange processes commonly observed for coordinated water molecules in solution.

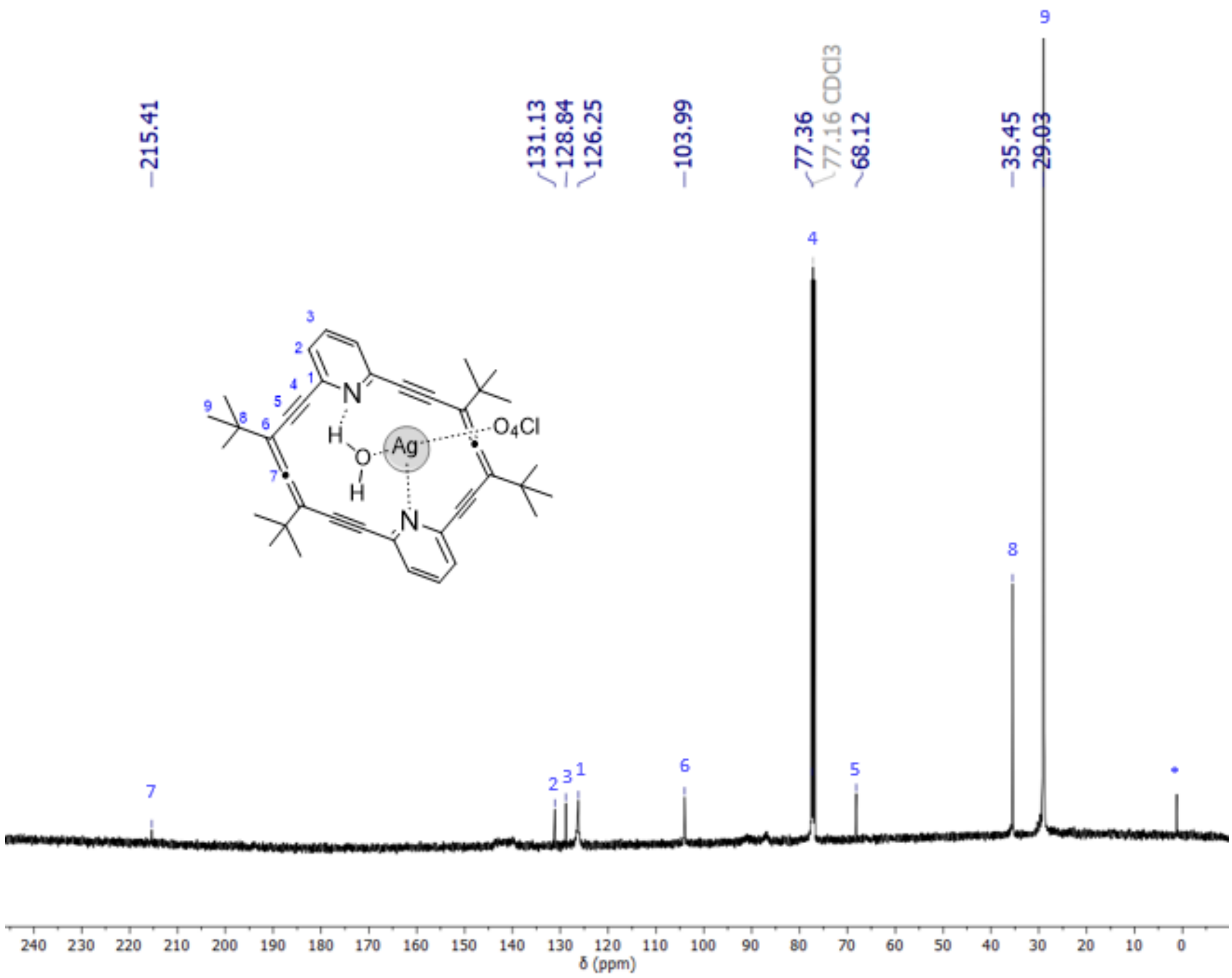

Figure S1b. *¹³C NMR (100 MHz) spectrum of complex* ***1a*** *in* $CDCl_3$*. Signals marked with an asterisk (*) correspond to impurities.*

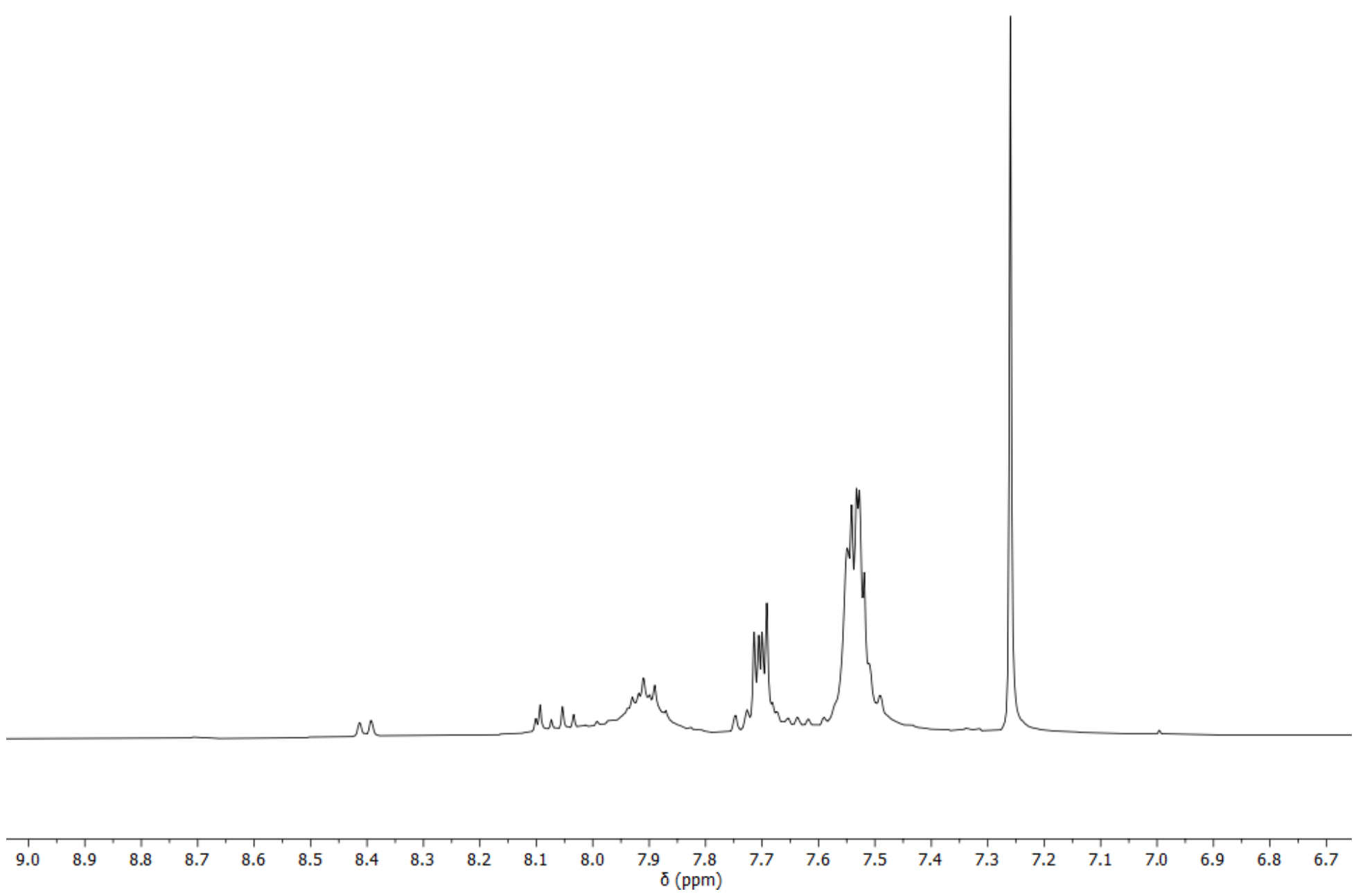

Figure S1c. *Expanded region of the ¹H NMR (400 MHz) spectrum obtained after dissolution of single crystals of complex* ***1b*** *in* $CDCl_3$*.*

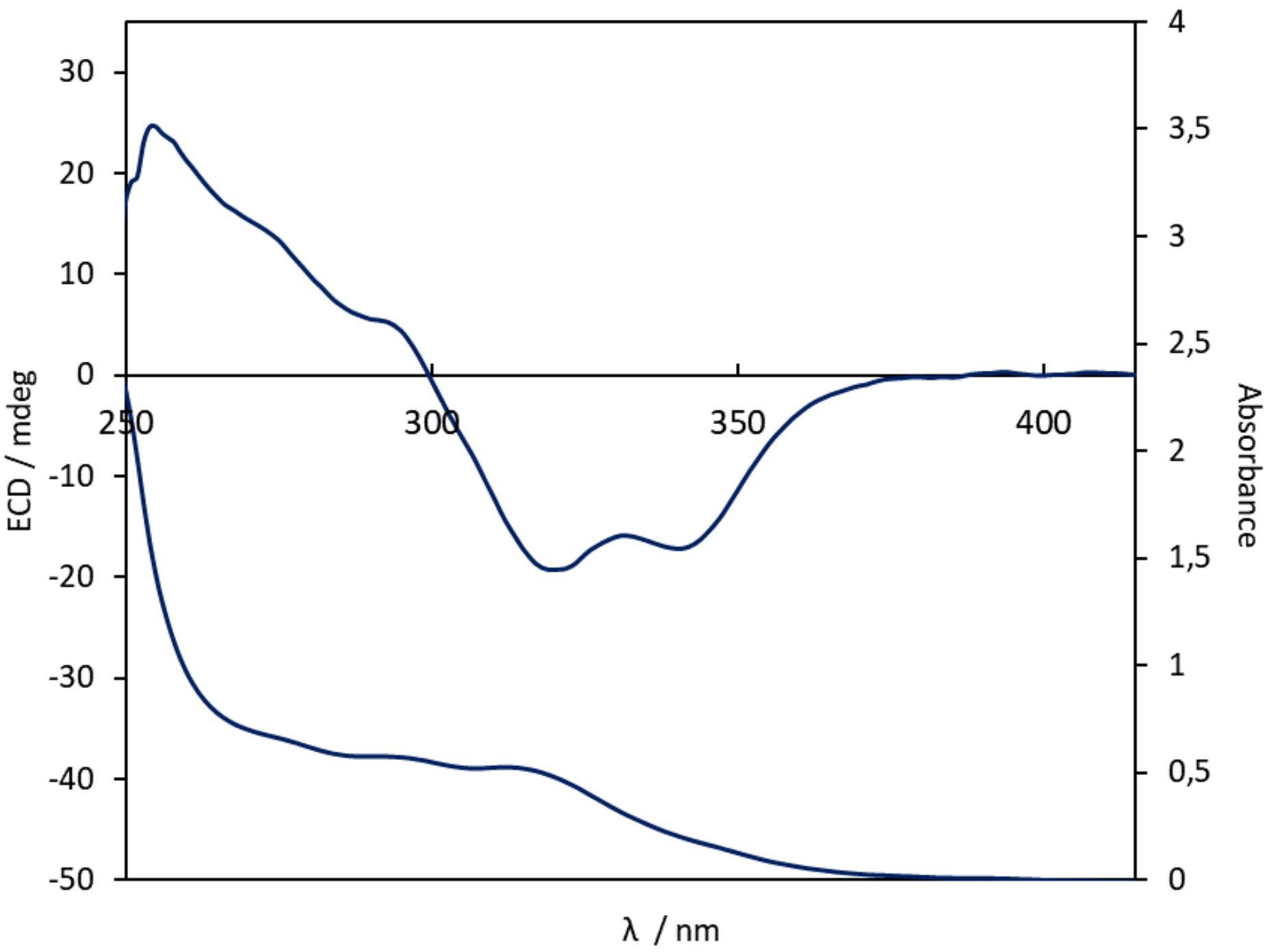

Figure S2a. *ECD (top) and UV-vis (bottom) spectra of (P$_2$)-**1a** (1.50 x 10-5 M in CHCl3:MeOH (95:5)*

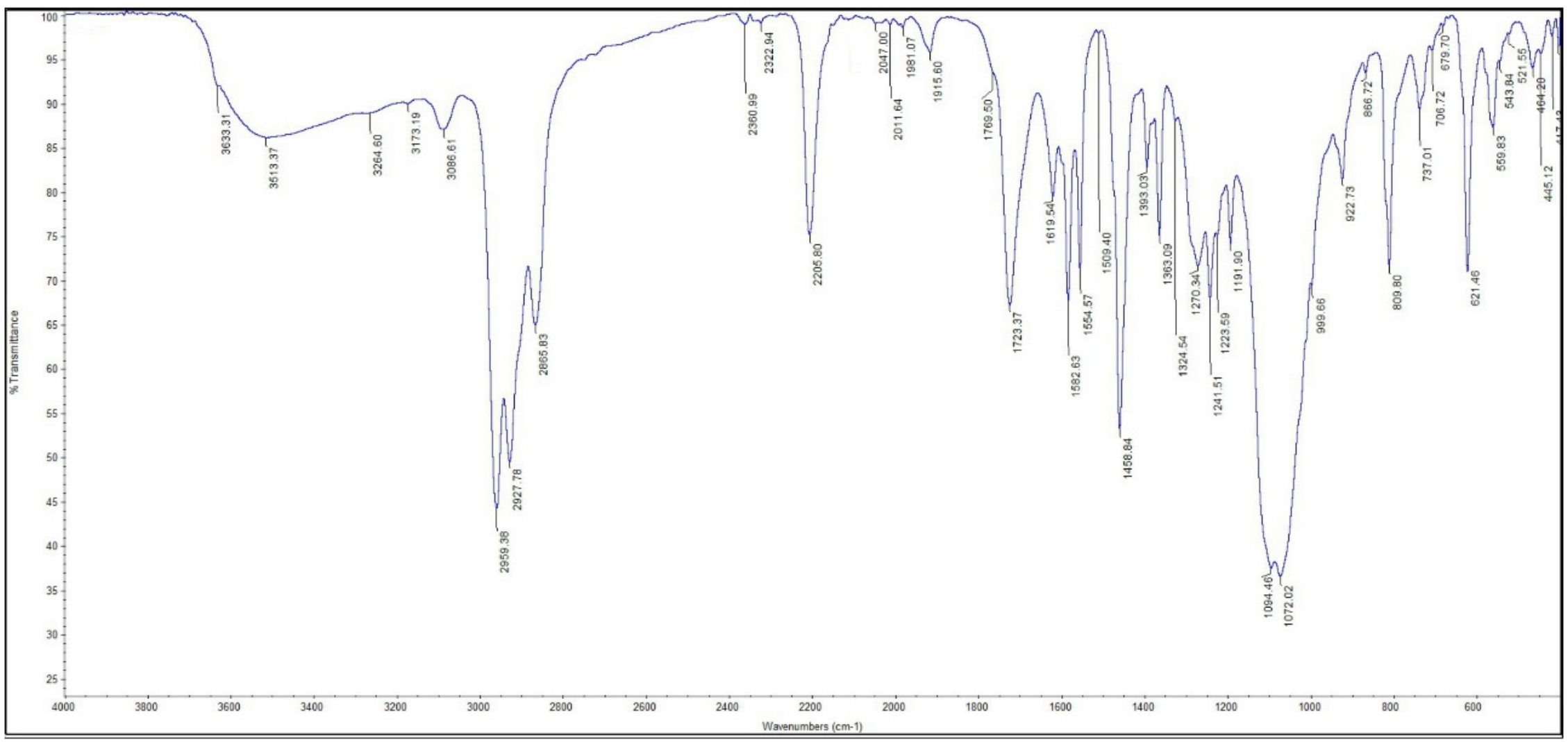

Figure S2b. *IR spectra of complex **1a.***

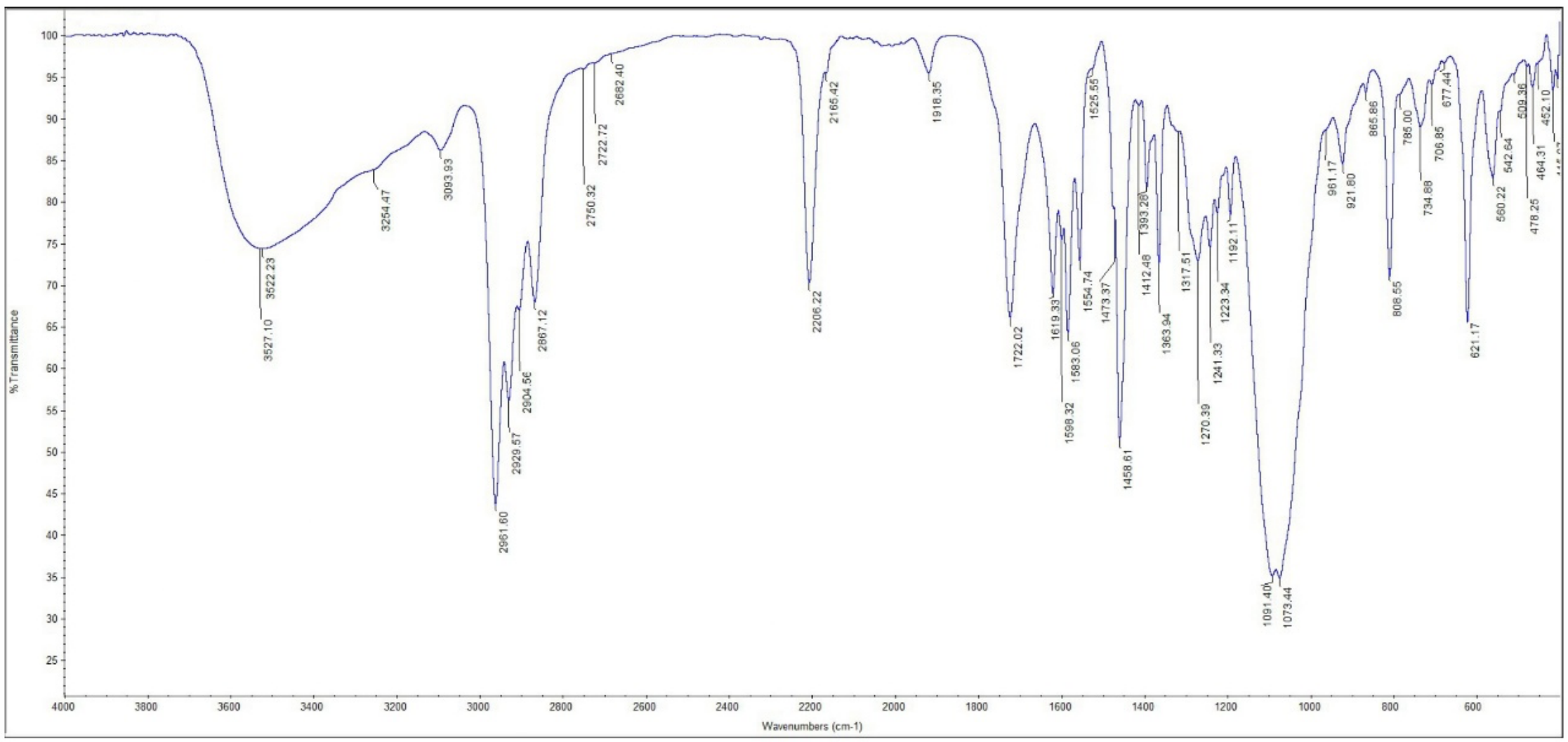

Figure S2c. *IR spectra of complex* ***1b.***

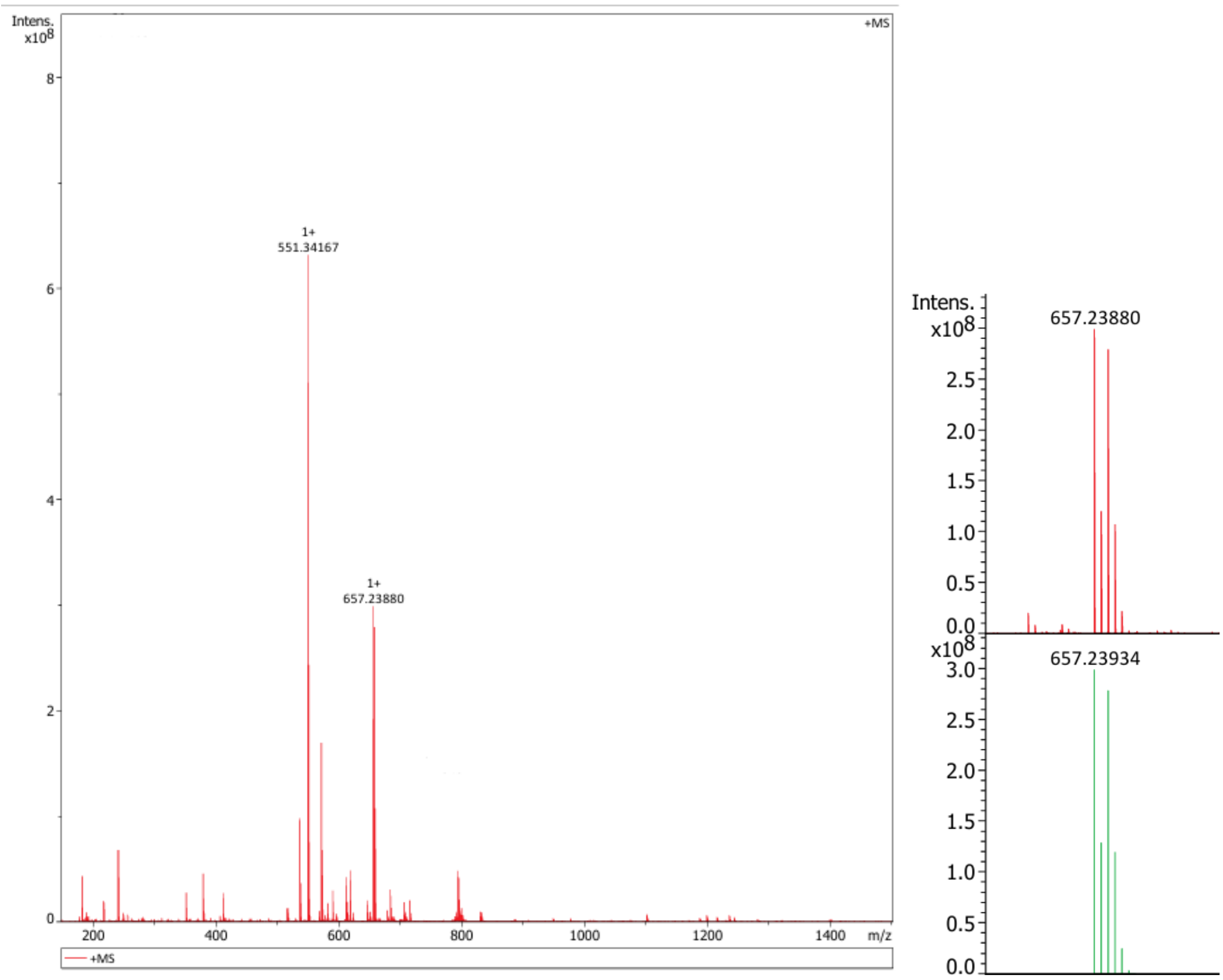

Figure S3. HRMS-ESI *m/z and isotopic pattern calcd for $C_{40}H_{42}N_2Ag^+$ (green); found (red). The signal at m/z = 551.34167 is assigned to the protonated free ligand ($C_{40}H_{43}N_2^+$).*

2.1. Crystallographic Data

Complex **1a**

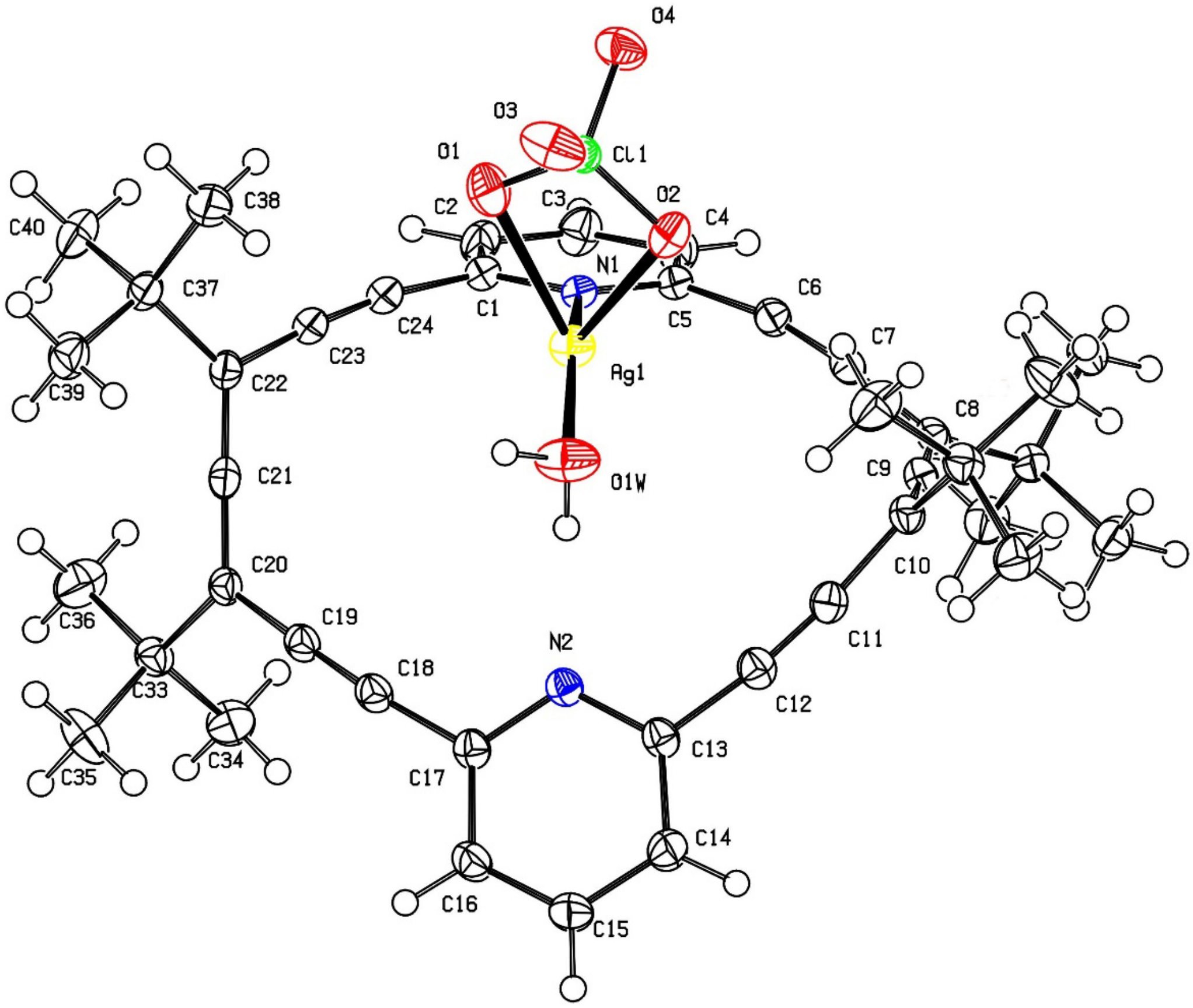

Figure S4. *Thermal ellipsoid plot for **1a** x-ray structure (50% probability).*

Table S1. Crystal data and structure refinement for **1a**.

Empirical formula	$C_{40}H_{44}AgClN_2O_5$	
Formula weight	776.09	
Temperature	100.00 K	
Wavelength	0.71073 Å	
Crystal system	Triclinic	
Space group	P-1	
Unit cell dimensions	a = 10.8304(5) Å	α= 115.413(2)°.
	b = 13.9238(8) Å	β= 102.591(2)°.
	c = 15.2457(8) Å	γ = 98.368(2)°.
Volume	1950.69(18) Å^3	
Z	2	
Density (calculated)	1.321 Mg/m^3	
Absorption coefficient	0.628 mm^{-1}	
F(000)	804	

Crystal size	0.178 x 0.171 x 0.119 mm^3
Theta range for data collection	2.002 to 28.337°.
Index ranges	-14<=h<=14, -18<=k<=18, -20<=l<=20
Reflections collected	113143
Independent reflections	9725 [R(int) = 0.0471]
Completeness to theta = 25.242°	99.9 %
Absorption correction	Semi-empirical from equivalents
Max. and min. transmission	0.2627 and 0.2192
Refinement method	Full-matrix least-squares on F^2
Data / restraints / parameters	9725 / 0 / 454
Goodness-of-fit on F^2	1.077
Final R indices [I>2sigma(I)]	R_1 = 0.0335, wR2 = 0.0901
R indices (all data)	R_1 = 0.0365, wR2 = 0.0920
Extinction coefficient	n/a
Largest diff. peak and hole	1.919 and -0.937 e.$Å^{-3}$

The silver atom is tetra coordinated, since it is bonded to a pyridinic nitrogen atom of the macrocycle, a water molecule and two oxygen atoms of the perchlorate anion. Besides, an intramolecular hydrogen bond, involving the water molecule as donor and the nitrogen atom of the second pyridine ring as acceptor, stabilises the formed complex (**Table S2**).

The nature of the complexes allows the formation self-assembled architectures by non-classical hydrogen bonds (**Table S2**) and O-H···π interactions (**Table S3**). Initially, dimers are formed by O-H···π interactions that establish coordinated water molecules with pyridine rings of neighbouring complexes (**Figure S5**, **Table S3**). Furthermore, the dimers are connected by non-classical hydrogen bonds, involving C-H groups of pyridine rings as donor and oxygen atoms of perchlorate anions as acceptors, forming a 2D (**Figure S6**). Finally, a 3D network is formed by additional hydrogen bonds (involving a methyl group), joining the 2D organizations (**Figure S7**).

Table S2. Hydrogen bond parameters (Å, °)

D-H···A	d(D-H)	d(H···A)	d(D···A)	∠(DHA)
O1w-H1wa···N2	0.8701(17)	1.9489(16)	2.753(2)	153.11(12)
C2-H2···O3_$1	0.95	2.47	3.194(3)	133.1
C4-H4···O2_$2	0.95	2.59	3.422(3)	146.1
C4-H4···O4_$2	0.95	2.47	3.367(3)	158.0
C40-H40a···O3_$3	0.98	2.49	3.442(3)	163.4

Symmetry codes: **$1** -1+x, y, z; **$2** 1-x, 1-y,- z; **$3** 2-x, 2-y, 1-z

Table S3. O-H···π interaction parameters (Å, °)

C-H···π	d[H···Cg(J)]	d[H···⊥P(J)]	γ	∠[C-H···Cg(J)]	d[C···Cg(J)]
O1w-H1w···Cg(1)_$4	2.95	2.66	25.73	149	3.723(2)

Cg(1): N2-C13-C14-C15-C16-C17; **$4** 2-x, 1-y, 1-z

Cg(J): Center of gravity of ring J.

d[H···Cg(J)]: Distance of H atom to Cg(J)

d[H···⊥P(J)]: Perpendicular distance of H atom to ring plane J.

γ: Angle between Cg(J)-H vector and ring J vector.

∠[C-H···Cg(J)]: C-H-Cg(J) angle.

d[C···Cg(J)]: Distance of C atom to Cg(J).

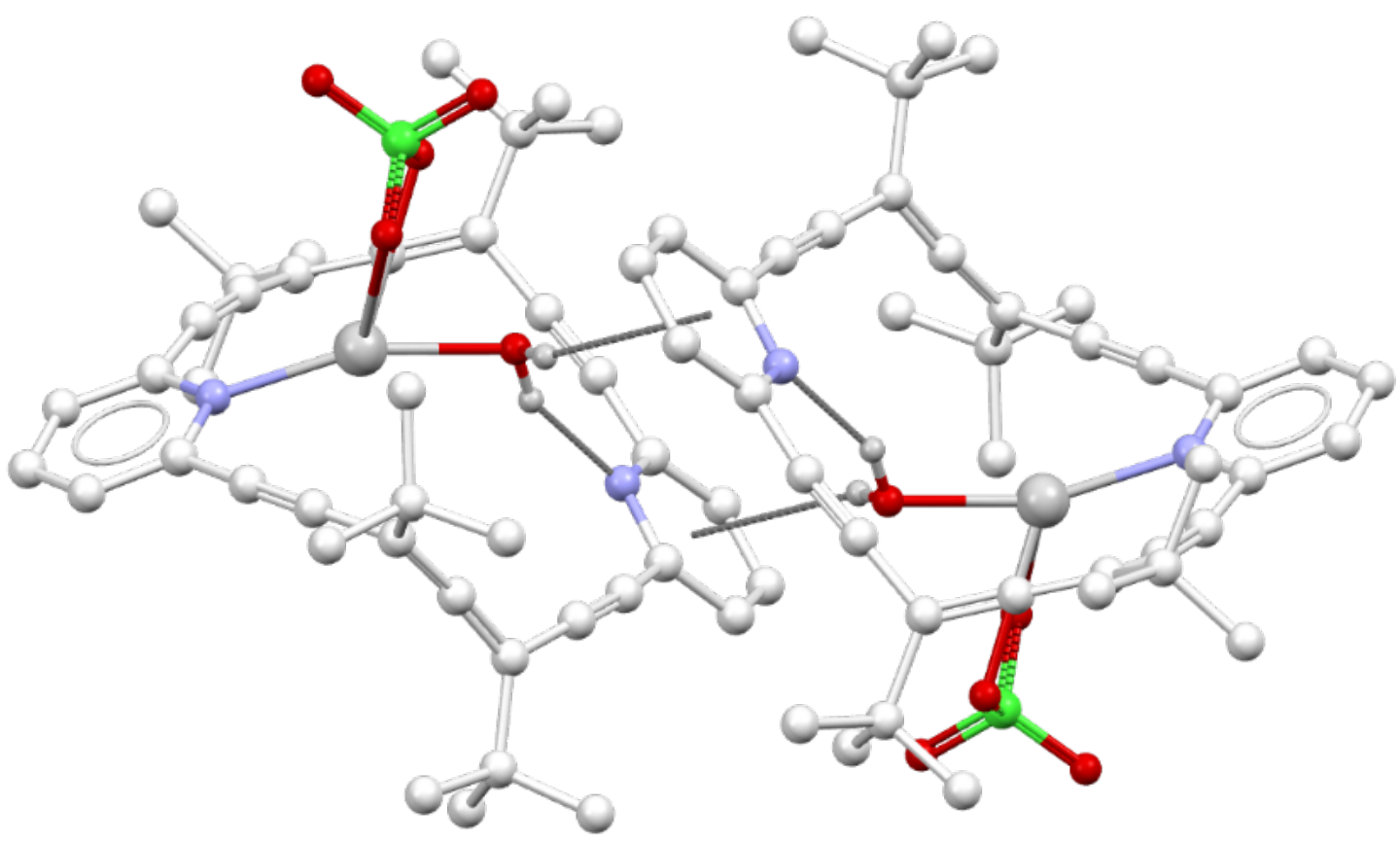

Figure S5. *Representation of **1a** racemic dimer, some hydrogen atoms omitted for clarity.*

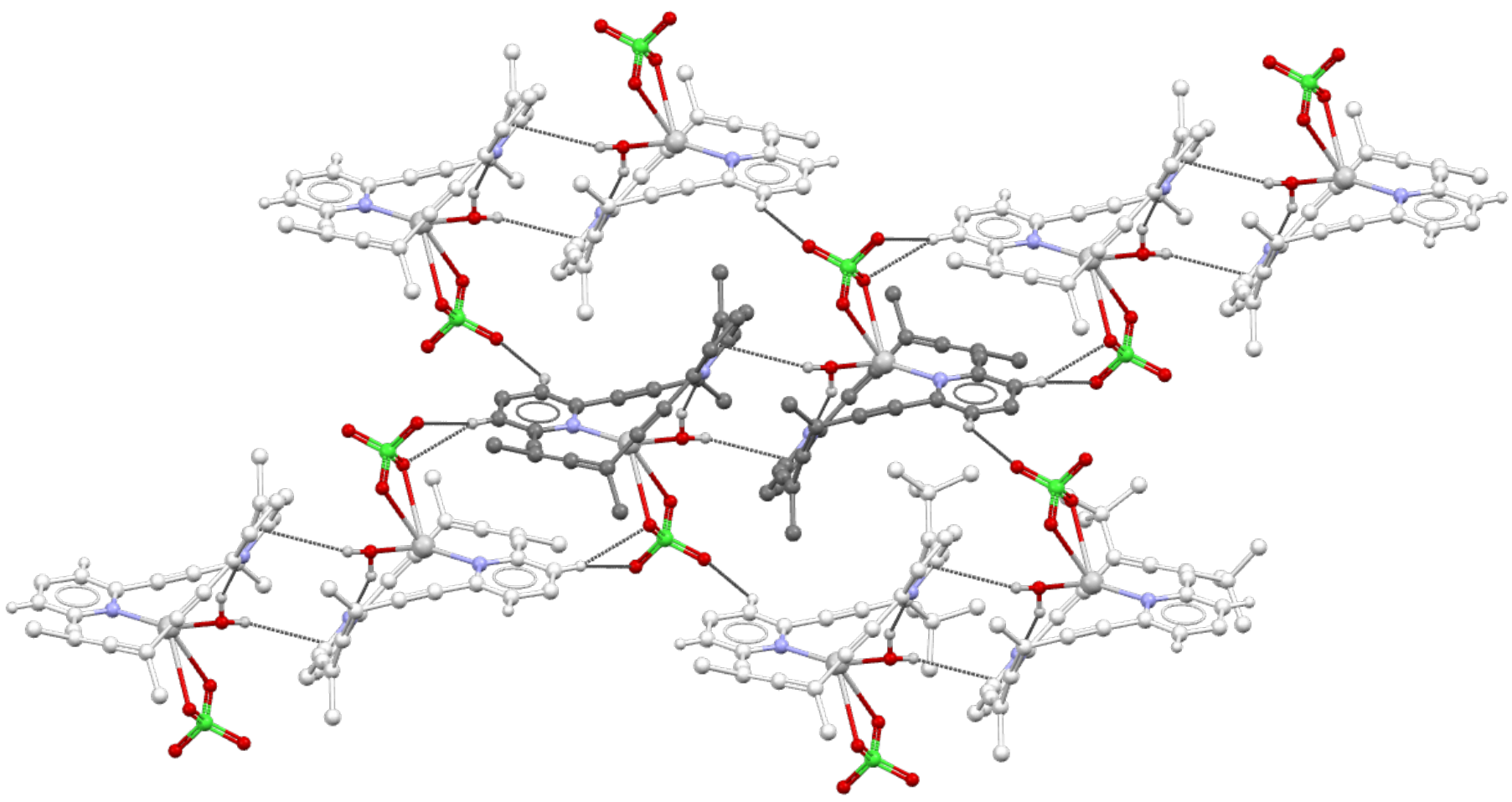

Figure S6. *Representation of 2D organisation, some hydrogen atoms and methyl groups omitted for clarity.*

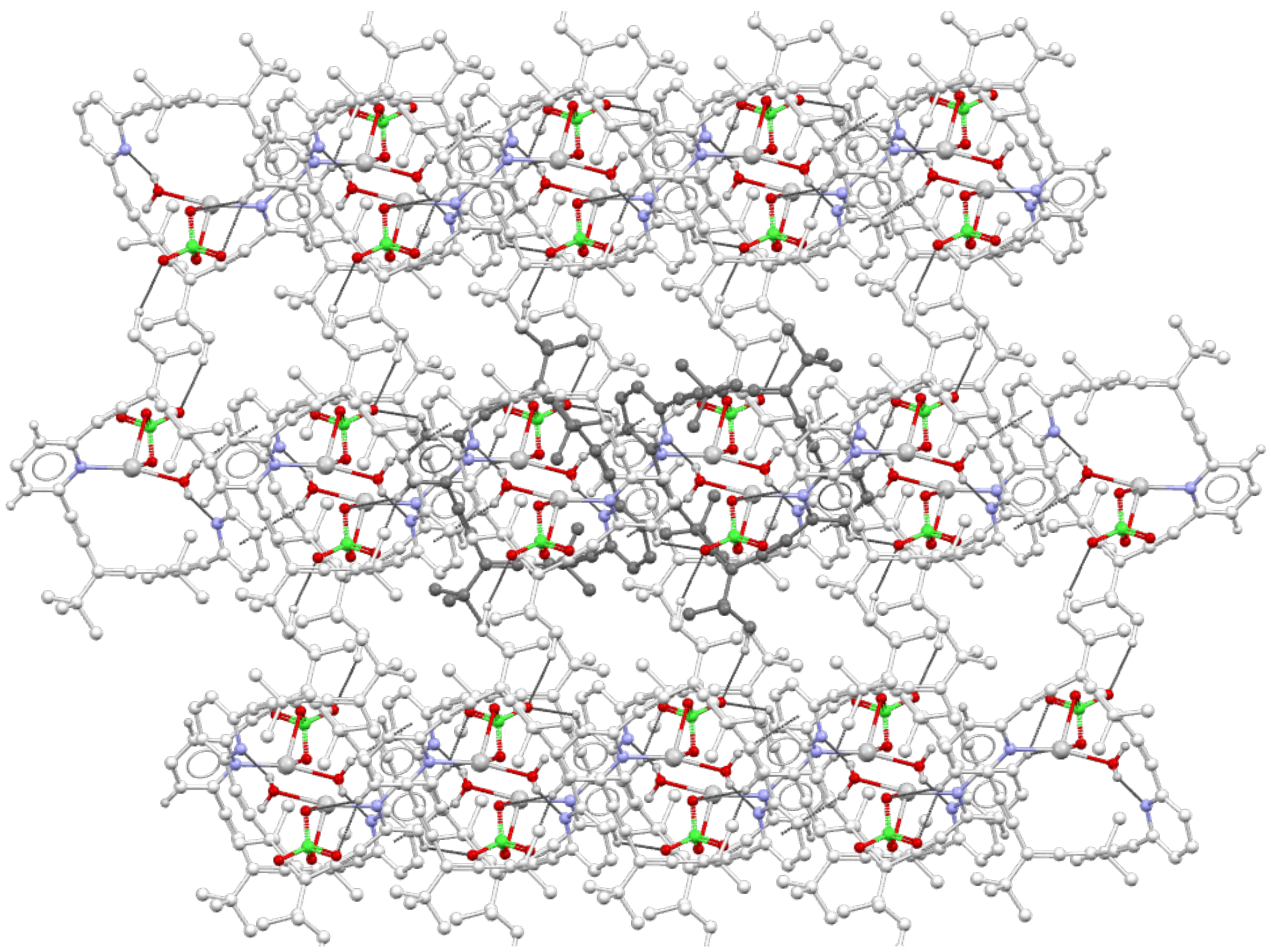

Figure S7. *Representation of final 3D network, some hydrogen atoms omitted for clarify.*

Complex **1b**

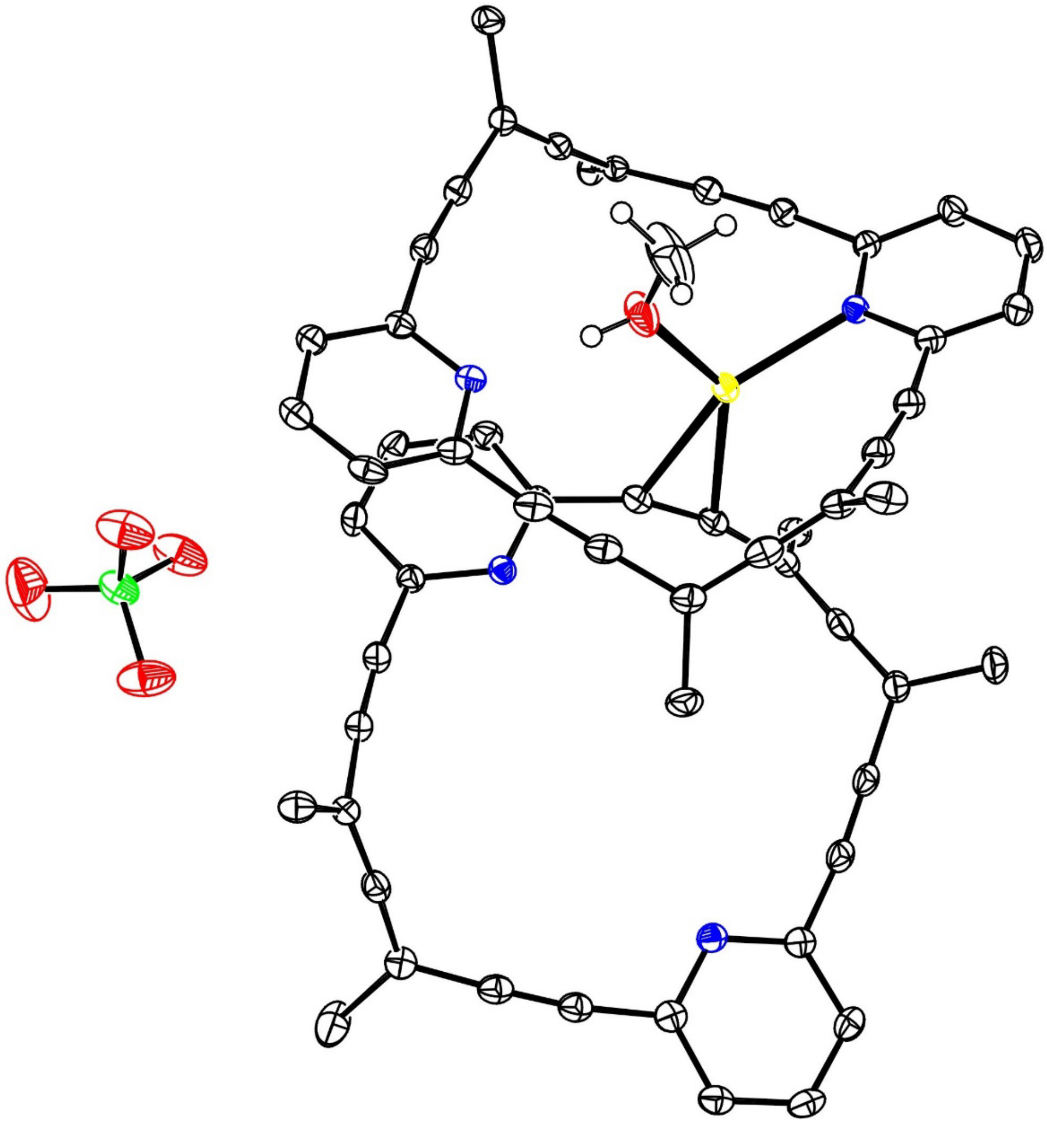

Figure S8. *Thermal ellipsoid plot of the X-ray crystal structure of* **1b** *shown at 50% probability. Tert-Butyl groups are omitted for clarity.*

Table S4. Crystal data and structure refinement for **1b.**

Empirical formula	$C_{81}H_{88}AgClN_4O_5$	
Formula weight	1340.87	
Temperature	100.00 K	
Wavelength	0.71073 Å	
Crystal system	Monoclinic	
Space group	P 2_1/c	
Unit cell dimensions	a = 23.3024(15) Å	α= 90°.
	b = 17.8357(12) Å	β= 99.485(2)°.
	c = 18.1836(12) Å	γ = 90°.
Volume	7454.1(9) $Å^3$	
Z	4	
Density (calculated)	1.195 Mg/m^3	
Absorption coefficient	0.358 mm^{-1}	
F(000)	2824	
Crystal size	0.149 x 0.093 x 0.089 mm^3	
Theta range for data collection	1.926 to 26.427°.	
Index ranges	-28<=h<=29, -22<=k<=22, -22<=l<=22	
Reflections collected	167170	
Independent reflections	15284 [R(int) = 0.0679]	
Completeness to theta = 25.242°	100.0 %	
Absorption correction	Semi-empirical from equivalents	
Max. and min. transmission	0.7385 and 0.6313	
Refinement method	Full-matrix least-squares on F^2	
Data / restraints / parameters	15284 / 39 / 871	
Goodness-of-fit on F^2	1.050	
Final R indices [I>2sigma(I)]	R_1 = 0.0353, wR2 = 0.0820	
R indices (all data)	R_1 = 0.0450, wR2 = 0.0882	
Extinction coefficient	n/a	
Largest diff. peak and hole	0.538 and -0.484 e.$Å^{-3}$	

Table S5. Comparison of Key Metric Distances in Complexes **1a** and **1b**

Structural Parameter	**1a** (Å)	**1b** (Å)
Ag···N	2.1650(16)	2.2358(16)
Ag···O	2.1895(17)	2.2104(16)
N···HO	1.9489(16)	1.9239(16)
Ag···C≡C	-	2.3085(19), 2.2469(19)

3. ECD Titration

A known amount of **L** (5 mg) was accurately weighed using an analytical balance and dissolved in a known volume of chloroform ($CHCl_3$). The solution was brought to volume using appropriate volumetric glassware. This stock solution was then diluted to obtain a final concentration of 1.5 × 10^{-5} M in chloroform. In parallel, a concentrated solution of $AgClO_4$ in methanol was prepared gravimetrically. Aliquots of this solution were incrementally added to the ligand-containing solution using RAININ Pos-D positive displacement micropipettes, such that the final methanol content in the chloroform solution did not exceed 5% v/v. The titration was repeated to ensure reproducibility. The data were fitted to a 1:1 binding model using the **Bindfit** software.[12]

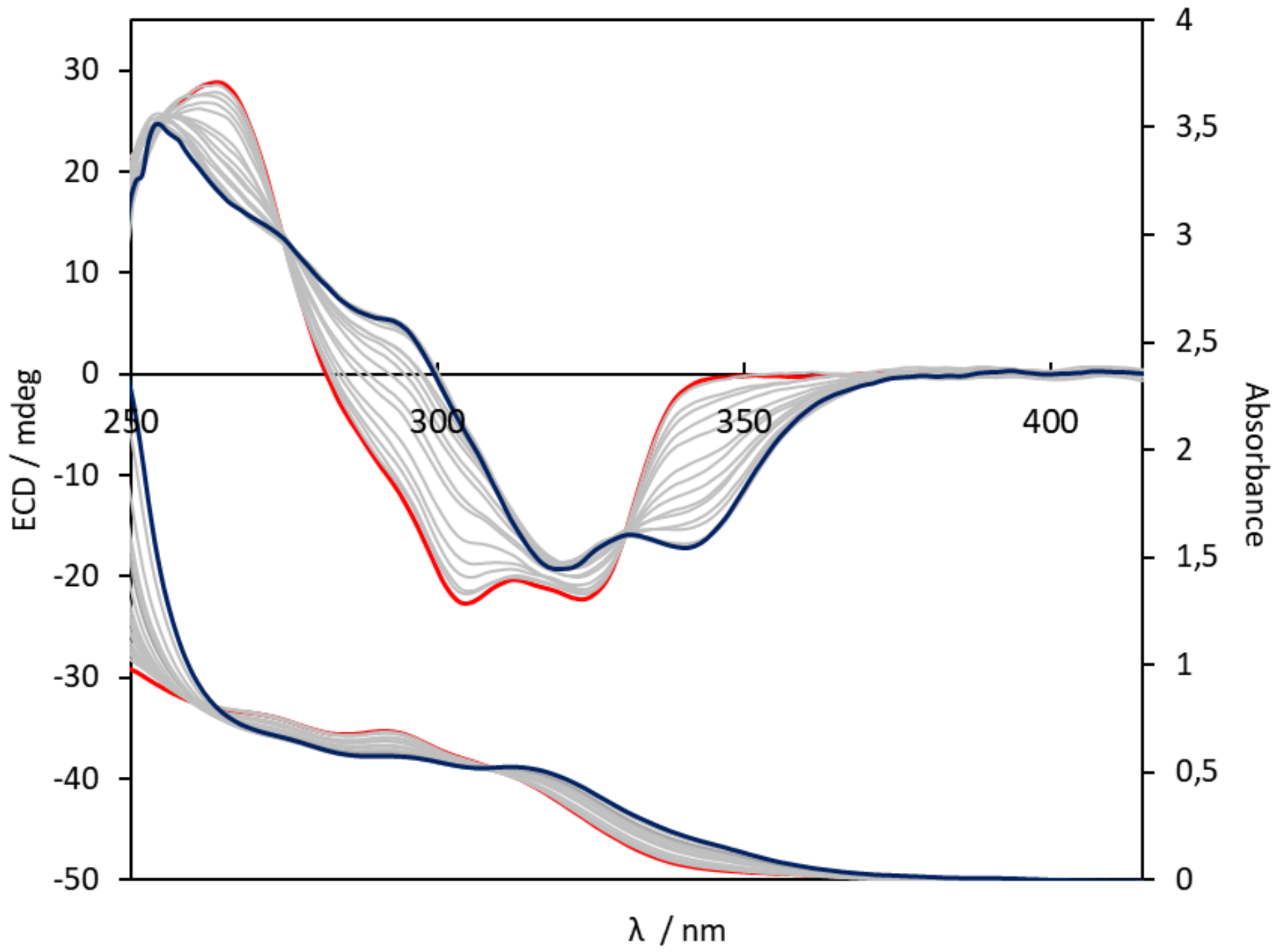

Figure S9. *Change of the ECD (top) and UV/Vis spectra (bottom) of (P_2)-**L** (1.5 x 10^{-5}M) upon addition of $AgClO_4$ ($CHCl_3$:MeOH (95:5). Red line: Original signal before adding $AgClO_4$. Blue line: ECD signal at the final saturation value. Grey lines: Changes with increasing amounts of $AgClO_4$.*

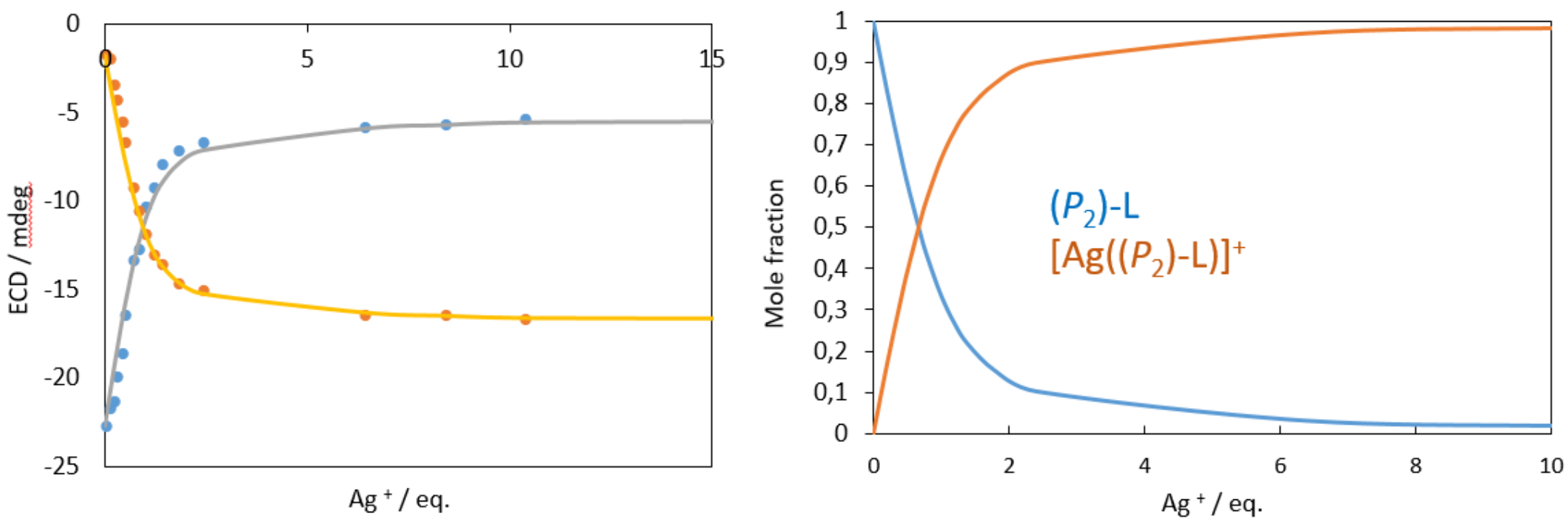

Figure S10. *Mathematical fitting of experimental data presented in* ***Figure S9*** *for the complexation of $AgClO_4$ by ligand (P_2)-**L** to a 1:1 stoichiometry, along with the corresponding species balance. On the left, the orange data points correspond to the ECD changes monitored at 305 nm, while the blue data points correspond to those recorded at 340 nm.*

4. Computational model of the 1a → 1b transformation

To evaluate the thermodynamic feasibility of the simplified transformation shown in **Figure S11**, the Gibbs free energy change (ΔG) was calculated according to the following equation:

$$\Delta G = \sum G_{products} - \sum G_{reactants}$$

The energy of each reactant and product was calculated independently at the same level of theory. Both reactant and product species were fully optimized using Gaussian package at the B3LYP-D3/ def2-TZVP level of theory.[13]

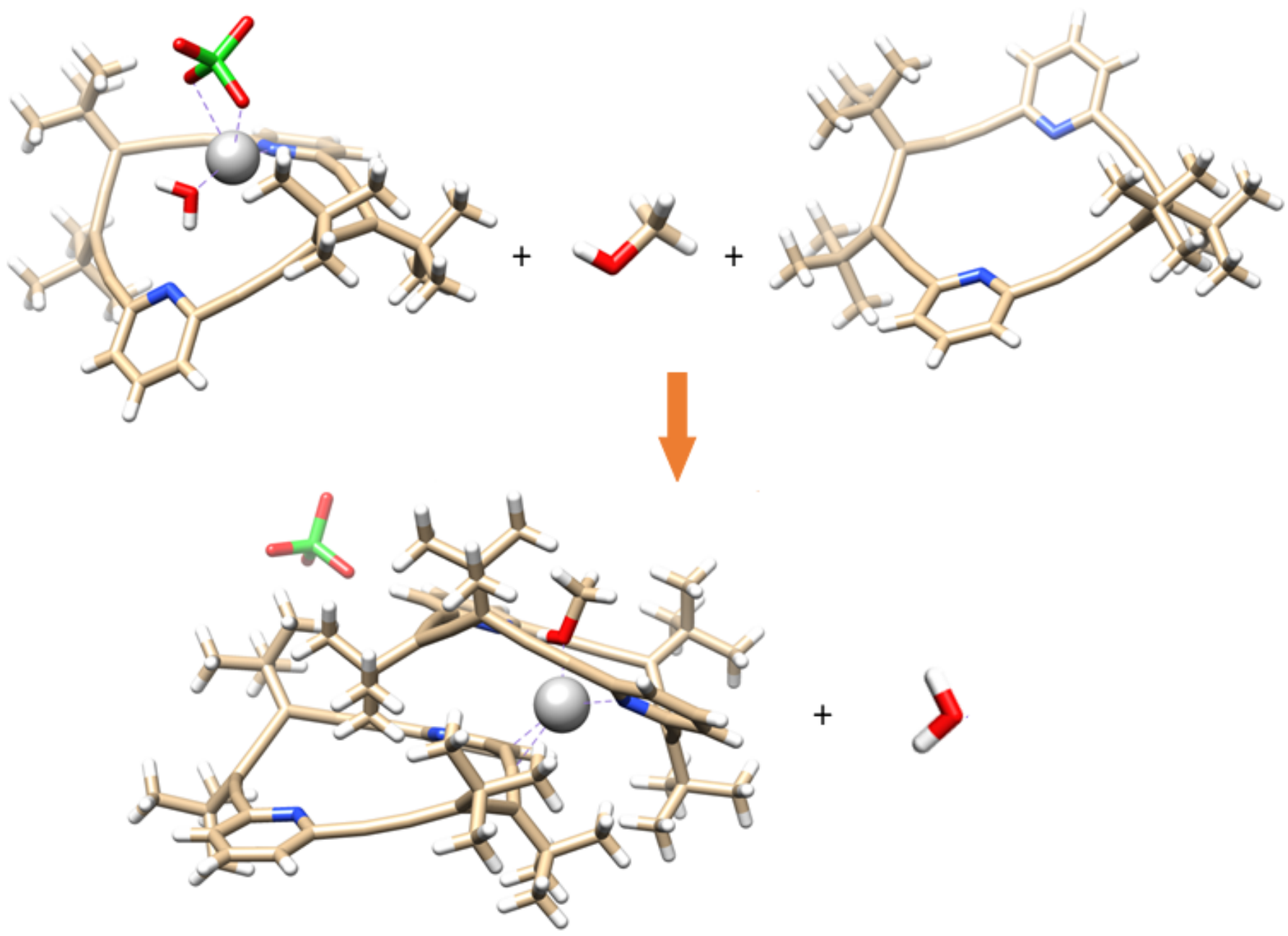

Figure S11. *Simplified computational model of the* ***1a*** *→* ***1b*** *transformation.*

5. Hole-electron analysis

The hole–electron analysis was performed using the **Multiwfn** software to investigate the origin of the main bands in the calculated **ECD spectrum** of **1a**. This method allows visualization of the spatial distribution of the hole and the excited electron during electronic transitions.[14] Drawings were made with VESTA.[15]

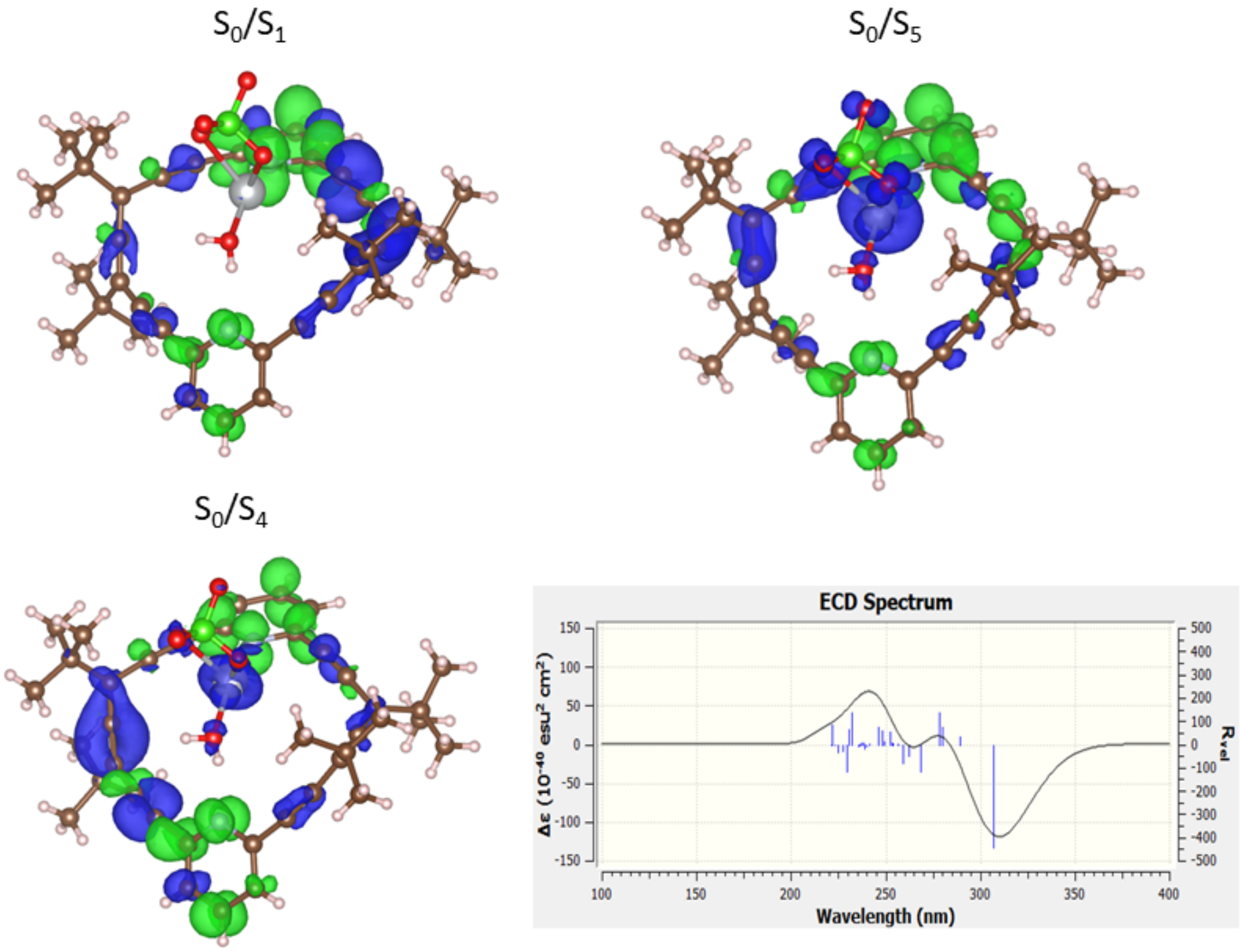

Figure S12. *Hole–electron analysis for the* $S_0 \rightarrow S_1$*,* $S_0 \rightarrow S_4$*, and* $S_0 \rightarrow S_5$ *transitions, and the calculated ECD spectrum of complex* ***1a****.*

6. References

(1) APEX4 v.2022.1-1 (Bruker AXS Inc., 2022).

(2) SAINT v. 8.40B (Bruker AXS Inc., 2019).

(3) Krause, L.; Herbst-Irmer, R.; Sheldrick, G. M.; Stalke, D. Comparison of Silver and Molybdenum Microfocus X-Ray Sources for Single-Crystal Structure Determination. *J. Appl. Crystallogr.* **2015**, *48* , 3–10.

(4) Sheldrick, G. M. SHELXT - Integrated Space-Group and Crystal-Structure Determination. *Acta Crystallogr. Sect. A Found. Crystallogr.* **2015**, *71* (1), 3–8.

(5) Sheldrick, G. M. Crystal Structure Refinement with SHELXL. *Acta Crystallogr. Sect. C Struct. Chem.* **2015**, *71* , 3–8.

(6) Dolomanov, O. V.; Bourhis, L. J.; Gildea, R. J.; Howard, J. A. K.; Puschmann, H. OLEX2: A Complete Structure Solution, Refinement and Analysis Program. *J. Appl. Crystallogr.* **2009**, *42* , 339–341.

(7) Yanai, T.; Tew, D. P.; Handy, N. C. A new hybrid exchange–correlation functional using the Coulomb-attenuating method (CAM-B3LYP). *Chem. Phys. Lett.,* **2004**, 393, 51-57.

(8) Weigend, F.; Ahlrichs, R. *Balanced Basis Sets of Split Valence, Triple Zeta Valence and Quadruple Zeta Valence Quality for H to Rn: Design and Assessment of Accuracy*. *Phys. Chem. Chem. Phys*. **2005**, *7*, 3297–3305.

(9) Pritchard, B. P.; Altarawy,D.; Didier, B.; Gibson, T. D.; Windus, T. L. *A New Basis Set Exchange: An Open, Up-to-date Resource for the Molecular Sciences Community. J. Chem. Inf. Model.* **2019**, *59(11)*, 4814-4820.

(10) Marenich, A. V.; Cramer, C. J.; Truhlar, D. G. Universal Solvation Model Based on Solute Electron Density and on a Continuum Model of the Solvent Defined by the Bulk Dielectric Constant and Atomic Surface Tensions. *J. Phys. Chem. B.,* **2009**, 113, 6378-6396.

(11) Álvarez-García, J.; Rubio-Pisabarro, V.; García-Río, L.; Cid, M.M. Deciphering the degree of proton-transfer in pyrido-cyclophanes by chiroptical outcomes in non-aqueous solvents. *Org. Chem. Front.,* **2023**, 10, 5435–5442.

(12) Supramolecuar.org BindFit v0.5; http://supramolecular.org/. Accessed: June 4, 2026.

(13) Grimme, S.; Antony, J.; Ehrlich, S.; Krieg, H. A Consistent and Accurate *Ab Initio* Parametrization of Density Functional Dispersion Correction (DFT-D) for the 94 Elements H-Pu. *J. Chem. Phys.* **2010**, *132*, 154104.

(14) Lu, T.; Chen, F. Multiwfn: A Multifunctional Wavefunction Analyzer. *J. Comput. Chem*. **2012**, *33*, 580–592.

(15) Momma, K.; Izumi, F. VESTA: A Three-Dimensional Visualization System for Electronic and Structural Analysis. *J. Appl. Crystallogr.* **2008**, *41*, 653–658.
